# Interventions on gender equity in the workplace: a scoping review

**DOI:** 10.1186/s12916-024-03346-7

**Published:** 2024-04-05

**Authors:** Andrea C. Tricco, Amanda Parker, Paul A. Khan, Vera Nincic, Reid Robson, Heather MacDonald, Rachel Warren, Olga Cleary, Elaine Zibrowski, Nancy Baxter, Karen E. A. Burns, Doug Coyle, Ruth Ndjaboue, Jocalyn P. Clark, Etienne V. Langlois, Sofia B. Ahmed, Holly O. Witteman, Ian D. Graham, Wafa El-Adhami, Becky Skidmore, France Légaré, Janet Curran, Gillian Hawker, Jennifer Watt, Ivy Lynn Bourgeault, Jeanna Parsons Leigh, Karen Lawford, Alice Aiken, Christopher McCabe, Sasha Shepperd, Reena Pattani, Natalie Leon, Jamie Lundine, Évèhouénou Lionel Adisso, Santa Ono, Linda Rabeneck, Sharon E. Straus

**Affiliations:** 1 Li Ka Shing Knowledge Institute, St. Michael’s Hospital, Unity Health Toronto, 209 Victoria Street, 7th Floor, East Building, Toronto, ON M5B 1T8 Canada; 2 Centre for Health Policy and Management, School of Medicine, Trinity College Dublin, Dublin, Ireland; 3 Faculty of Health Studies, Western University, London, Canada; 4 Melbourne School of Population and Global Health, University of Melbourne, Melbourne, Victoria Australia; 5 Interdepartmental Division of Critical Care Medicine, University of Toronto, Toronto, Canada; 6 School of Epidemiology and Public Health, University of Ottawa, Ottawa, Canada; 7 École de Travail Social, Université de Sherbrooke, Québec, (Québec) Canada; 8 Department of Medicine, University of Toronto, Toronto, Canada; 9 Partnership for Maternal, Newborn and Child Health (PMNCH), World Health Organization (WHO), Geneva, Switzerland; 10 Cumming School of Medicine, University of Calgary, Alberta, Canada; 11 Department of Family and Emergency Medicine, Faculty of Medicine, Université Laval, Quebec City, Canada; 12 Centre for Implementation Research, Ottawa Hospital Research Institute, Ottawa, Canada; 13 Science in Australia Gender Equity Limited, Greenway, Australia; 14 Independent Information Specialist, Ottawa, Canada; 15 School of Nursing, Faculty of Health, Dalhousie University, Halifax, Canada; 16 Division of Rheumatology, Department of Medicine, University of Toronto, Toronto, Canada; 17 University of Ottawa, Ottawa, Canada; 18 School of Health Administration, Faculty of Health, Dalhousie University, Halifax, Canada; 19 Department of Gender Studies, Haudenosaunee and Anishinaabek Territories, Queen’s University, Settlement of Kingston, Canada; 20 Research and Innovation, Dalhousie University, Halifax, Canada; 21 Institute of Health Economics, Edmonton, Canada; 22 Nuffield Department of Population Health, University of Oxford, Richard Doll Building, Old Road Campus, Oxford, UK; 23 Department of Medicine, Division of Internal Medicine, St Michael’s Hospital, Unity Health Toronto, Toronto, Canada; 24 Health Systems Research Unit, South African Medical Research Council, Cape Town, South Africa; 25 Department of Social and Preventive Medicine, Faculty of Medicine, Université Laval, Quebec City, Canada; 26 Molecular, Cellular, and Developmental Biology, University of Michigan, Ann Arbor, MI USA; 27 Dalla Lana School of Public Health, University of Toronto, Toronto, Canada

**Keywords:** Scoping review, Gender, Equity, Employment, Occupational health

## Abstract

**Background:**

Various studies have demonstrated gender disparities in workplace settings and the need for further intervention. This study identifies and examines evidence from randomized controlled trials (RCTs) on interventions examining gender equity in workplace or volunteer settings. An additional aim was to determine whether interventions considered intersection of gender and other variables, including PROGRESS-Plus equity variables (e.g., race/ethnicity).

**Methods:**

Scoping review conducted using the JBI guide. Literature was searched in MEDLINE, Embase, PsycINFO, CINAHL, Web of Science, ERIC, Index to Legal Periodicals and Books, PAIS Index, Policy Index File, and the Canadian Business & Current Affairs Database from inception to May 9, 2022, with an updated search on October 17, 2022. Results were reported using Preferred Reporting Items for Systematic Reviews and Meta-Analyses extension to scoping reviews (PRISMA-ScR), Sex and Gender Equity in Research (SAGER) guidance, Strengthening the Integration of Intersectionality Theory in Health Inequality Analysis (SIITHIA) checklist, and Guidance for Reporting Involvement of Patients and the Public (GRIPP) version 2 checklist.

All employment or volunteer sectors settings were included. Included interventions were designed to promote workplace gender equity that targeted: (a) individuals, (b) organizations, or (c) systems. Any comparator was eligible. Outcomes measures included any gender equity related outcome, whether it was measuring intervention effectiveness (as defined by included studies) or implementation. Data analyses were descriptive in nature. As recommended in the JBI guide to scoping reviews, only high-level content analysis was conducted to categorize the interventions, which were reported using a previously published framework.

**Results:**

We screened 8855 citations, 803 grey literature sources, and 663 full-text articles, resulting in 24 unique RCTs and one companion report that met inclusion criteria. Most studies (91.7%) failed to report how they established sex or gender. Twenty-three of 24 (95.8%) studies reported at least one PROGRESS-Plus variable: typically sex or gender or occupation. Two RCTs (8.3%) identified a non-binary gender identity. None of the RCTs reported on relationships between gender and other characteristics (e.g., disability, age, etc.). We identified 24 gender equity promoting interventions in the workplace that were evaluated and categorized into one or more of the following themes: (i) quantifying gender impacts; (ii) behavioural or systemic changes; (iii) career flexibility; (iv) increased visibility, recognition, and representation; (v) creating opportunities for development, mentorship, and sponsorship; and (vi) financial support. Of these interventions, 20/24 (83.3%) had positive conclusion statements for their primary outcomes (e.g., improved academic productivity, increased self-esteem) across heterogeneous outcomes.

**Conclusions:**

There is a paucity of literature on interventions to promote workplace gender equity. While some interventions elicited positive conclusions across a variety of outcomes, standardized outcome measures considering specific contexts and cultures are required. Few PROGRESS-Plus items were reported. Non-binary gender identities and issues related to intersectionality were not adequately considered. Future research should provide consistent and contemporary definitions of gender and sex.

**Trial registration:**

Open Science Framework https://osf.io/x8yae.

**Supplementary Information:**

The online version contains supplementary material available at 10.1186/s12916-024-03346-7.

## Summary box

### What is already known on this topic

- Our previous large scoping review of gender equity interventions within academic health research identified more than 560 studies published over 50 years, showing tremendous research interest in gender equity.

### What this study adds

- This study summarizes the evidence from extensive review and synthesis of randomized evidence on gender equity interventions within workplace settings and shows that such interventions largely succeed and elicit mostly positive conclusions across a variety of outcomes, such as improving academic productivity and increased self-confidence and self-esteem.
- Many different outcomes were used to examine the effectiveness of gender equity interventions, suggesting that standardized outcome measures are required that consider specific contexts and cultures.
- Equity variables beyond sex or gender, or occupation, such as race/ethnicity, religion and age, are underreported, and notably sex/gender is neither reliably defined, nor are definitions consistently provided. Sex/gender terminology is conflated, and intersectionality is rarely considered. More comprehensive reporting and standardization aligned with growing community expectations for a range of equity variables are needed.
- These results can be utilized by researchers, funders, peer reviewers, and journal editors to both enhance, and establish, consistent reporting of gender equity research. More importantly, the findings can be used to inform the development and implementation of interventions to enhance gender equity in the workplace.

## Background

Ahead of the 2023 International Women’s Day, the United Nations Secretary General stated that “gender equality is growing more distant with estimates from other organizations (UN Women) placing it 300 years away” [1]. This suggests that the United Nations Sustainable Development goal five to “achieve gender equality and empower all women and girls” is getting further out of reach [2]. Furthermore, a recent report (2022) from the Melinda French Gates Foundation estimated that it will be 100 years until gender equality is fully realized [3, 4]. If women were equal participants, it is estimated that the global economy would grow by almost US $30 trillion per year [5]. Women are being left behind in the workplace, and in vital sectors, including in science and technology [1]. Women are also under-represented in leadership positions with 70.0% of health worker jobs held by women, yet only 25.0% of senior leadership positions held by women [6]. Solutions are needed to address the observed gender gap [1].

Recently, we published a large scoping review, including more than 560 studies over a 50-year period, focused on examining gender equity within academic health research [7]. Most studies (65.0%) did not report how gender or sex were determined/defined or they interchanged/conflated the terms of sex and gender, and all studies classified gender as a binary variable [7]. Gender is a social construct and as such is constantly in flux. Gender encompasses concepts such as gender roles and gender identity, which are important to consider when we look at gender equity. Sex is a biological construct, which encompasses anatomy, physiology, genes, and hormones. Sex impacts how we are labeled in society, and in research, it is common to adopt a binary understanding of man/woman, which can compromise the validity and generalizability of findings [8]. In our previous research, only three studies mentioned the intersection of gender and other variables [7]. Few studies reported the PROGRESS-Plus equity variables (i.e., place of residence, race/ethnicity/culture/language, occupation, gender/sex, religion, education, socioeconomic status, or social capital) [9], such as race/ethnicity (11.4%), religion (0.2%), and age (7.3%) [7]. Our review concluded that interventions to achieve gender equity in academia and in all workplace settings that account for actual lived experience are required [7].

This scoping review sought to summarize the evidence from randomized controlled trials (RCTs) on gender equity interventions within any workplace setting. Scoping reviews provide a high-level summary of the evidence within a concept (here it is gender equity interventions) and are useful for highlighting definitions, characteristics, and factors related to that concept [10]. As such, additional objectives were to determine whether any interventions considered the intersection of gender and other variables [11, 12] and if any studies reported the PROGRESS-Plus equity variables [9]. A scoping review approach was used, as our research question was broad, and our goal was to identify and catalogue the evidence on workplace gender equity interventions from randomized trials [13].

## Methods

### Protocol

A protocol was developed using guidance on scoping review protocols [14, 15]. The JBI (formerly Joanna Briggs Institute) guidance for scoping reviews [13] informed the conduct of this scoping review. The protocol for this scoping review was registered with Open Science Framework (https://osf.io/x8yae). Team demographics and positionality are reported in the previous publication [7]. Prior to beginning this review, a self-reflective equity exercise was completed [16] to create an inclusive and respectful space for the team to openly share and contribute to the project. Knowledge users from multiple organizations engaged in all aspects of this scoping review. Review results are reported using all relevant reporting guidance: Preferred Reporting Items for Systematic Reviews and Meta-Analyses extension to scoping reviews (PRISMA-ScR [17]; Additional file 1: Appendix 1), Sex and Gender Equity in Research (SAGER) guidance [18] (Additional file 1: Appendix 2), Strengthening the Integration of Intersectionality Theory in Health Inequality Analysis (SIITHIA) checklist [19] (Additional file 1: Appendix 3), and Guidance for Reporting Involvement of Patients and the Public (GRIPP) version 2 checklist [20] (Additional file 1: Appendix 4).

### Literature search

The literature search was developed by an experienced librarian (BS) and peer-reviewed by another librarian using the Peer Review of Electronic Search Strategies (PRESS) checklist [21]. Electronic databases MEDLINE, Embase, PsycINFO, Cumulated Index to Nursing and Allied Health Literature (CINAHL), Web of Science, Education Resources Information Center (ERIC), Index to Legal Periodicals and Books, Public Affairs Information Service (PAIS) Index, Policy Index File, and the Canadian Business & Current Affairs Database were searched from inception to May 9, 2022. To ensure that all gender equity literature search terms were adequately captured, an updated literature search was executed on October 17, 2022, on all databases except for in MEDLINE, Embase, PsycINFO, and CINAHL. The literature search strategies for all databases can be found in Additional file 1: Appendix 5. Unpublished and grey literature was searched using the Canadian Agency for Drugs and Technologies in Health (CADTH)’s Grey Matters guidance [22]. A full list of grey literature sources is provided in Additional file 1: Appendix 6. Conference abstracts and dissertations identified through our literature search were screened for eligibility, and attempts were made to locate corresponding publications. Reference lists of all included trials and related reviews [7, 23–45] were manually scanned for additional trials of interest.

### Eligibility criteria

#### Population

Adults of any gender aged 18 years and above in any employment or volunteer sector, such as academia, government, education, or business.

#### Intervention

Any intervention designed to promote gender equity that targeted: (a) individuals (e.g., training in diversity, unconscious bias, mentorship, or coaching), (b) organizations (e.g., policies designed to address gender inequity, workplace code of conduct, or implementation of equity, and diversity and inclusion action plan at the government level), or (c) systems (e.g., legislation to publicly report salaries, legislation to mandate equitable representation on committees, or pay equity).

#### Comparator

Any comparator was eligible, including no comparator or usual practice.

#### Outcome

Any outcome related to gender equity, such as change in attitude, bias, and/or awareness.

#### Study designs

Only RCTs or quasi-randomized controlled trials were included.

#### Other

No restrictions were applied based on study year, language of dissemination, or study duration.

A screening form (presented in Additional file 1: Appendix 7) was developed based on pre-defined eligibility criteria. The reviewers completed a training exercise using 50 citations to ensure adequate agreement was achieved. After completing one training exercise (achieving 75.0% agreement), all remaining titles and abstracts identified in the search were screened independently by expert pairs of reviewers (AP, HM, OC, PAK, RW, RR, VN). Discrepancies were resolved by a third reviewer.

Similarly, a training exercise (Additional file 1: Appendix 8) was completed for screening of full-text articles, using 20 articles. Two training exercises were necessary (achieving 65.0% and 85.0% agreement, respectively). The screening form was then revised for clarity and full-text articles were assigned to independent pairs of reviewers (AP, HM, OC, PAK, RW, RR, VN). Discrepancies were consistently resolved by a third reviewer (AP). A glossary of key terms that guided the team is in Additional file 1: Appendix 9.

### Data abstraction

A data abstraction form (Additional file 1: Appendix 9) was created to capture data on the following items: study characteristics (e.g., country of conduct, country economy levels, settings), population characteristics (e.g., gender, sex, age), intervention characteristics (e.g., intersectionality, sample size, duration of intervention), and outcomes (e.g., culture change, number of publications). To capture outcomes relevant to equity, the PROGRESS-Plus criteria were used [9]. Additional relevant outcomes included intersectionality theory (defined as “an analytic framework and research paradigm that consider the ways in which connected systems and structures of power operate across time, place, and societal levels to construct intersecting social locations and identities (e.g., along axes such as race, gender, class, and sexual orientation, among others [19])), definitions (if any) of sex and gender by the authors, and changes in sexism, self advocacy, and financial autonomy. Full data abstraction was completed by independently by two reviewers (AP, VN, PAK, HC, RW, RR, and OC), with discrepancies solved by a third reviewer (AP).

### Analysis and presentation of results

Review findings were summarized descriptively using summary tables, figures, and text. As recommended in the JBI guide to scoping reviews [13], only high level content analysis was conducted to categorize the interventions, which were reported using a previously published framework [46]. Conclusion statements from each included trial were classified into one of four main categories: (positive, neutral, negative, and indeterminate [47]). The conclusion statements from the included articles were categorized by one team member (AP) and verified by another (ACT). When hypothesizing the benefit of an intervention (vs. a comparator), conclusion statements were classified as: positive (i.e., non-statistically significant positive, and statistically significant positive with an associated *P*-value < 0.05); neutral (effect size between 0.95 and 1.05 and the confidence interval (CI) crosses 1); negative, namely, there is an effect in favor of the nonintervention comparator (i.e., statistically significant negative with an associated *P*-value < 0.05, and non-statistically significant negative), or indeterminate (i.e., not able to judge; e.g., the article lists 10 primary outcomes, all of which have different results). Since this was a scoping review, a formal sex and gender-based analysis was not conducted in keeping with JBI guidance for scoping reviews [13].

### Patient and public involvement

A public partner, defined using the Canadian Institutes of Health Research glossary [48], was involved in this project from the outset (Additional file 1: Appendix 4). The public partner came from her lived experience as a woman in the workplace (EZ) and provided input and feedback on the protocol, title, and abstract screening form, full-text screening form, and final manuscript. The burden for the public partner was assessed from the outset to be no more than 2 h per month, which was agreed upon by the partner in advance. Our team uses a compensation policy that was co-produced by patient and public partners, policy-makers, healthcare providers, and researchers [49]. To support dissemination, the research team prepared and disseminated monthly progress reports to all authors for the project duration. We acknowledged our public partner’s contribution by including her as an author, and the team will involve the public partner in the development of the dissemination plan to access groups and forums the research team may not be aware of.

## Results

**Fig. 1:**
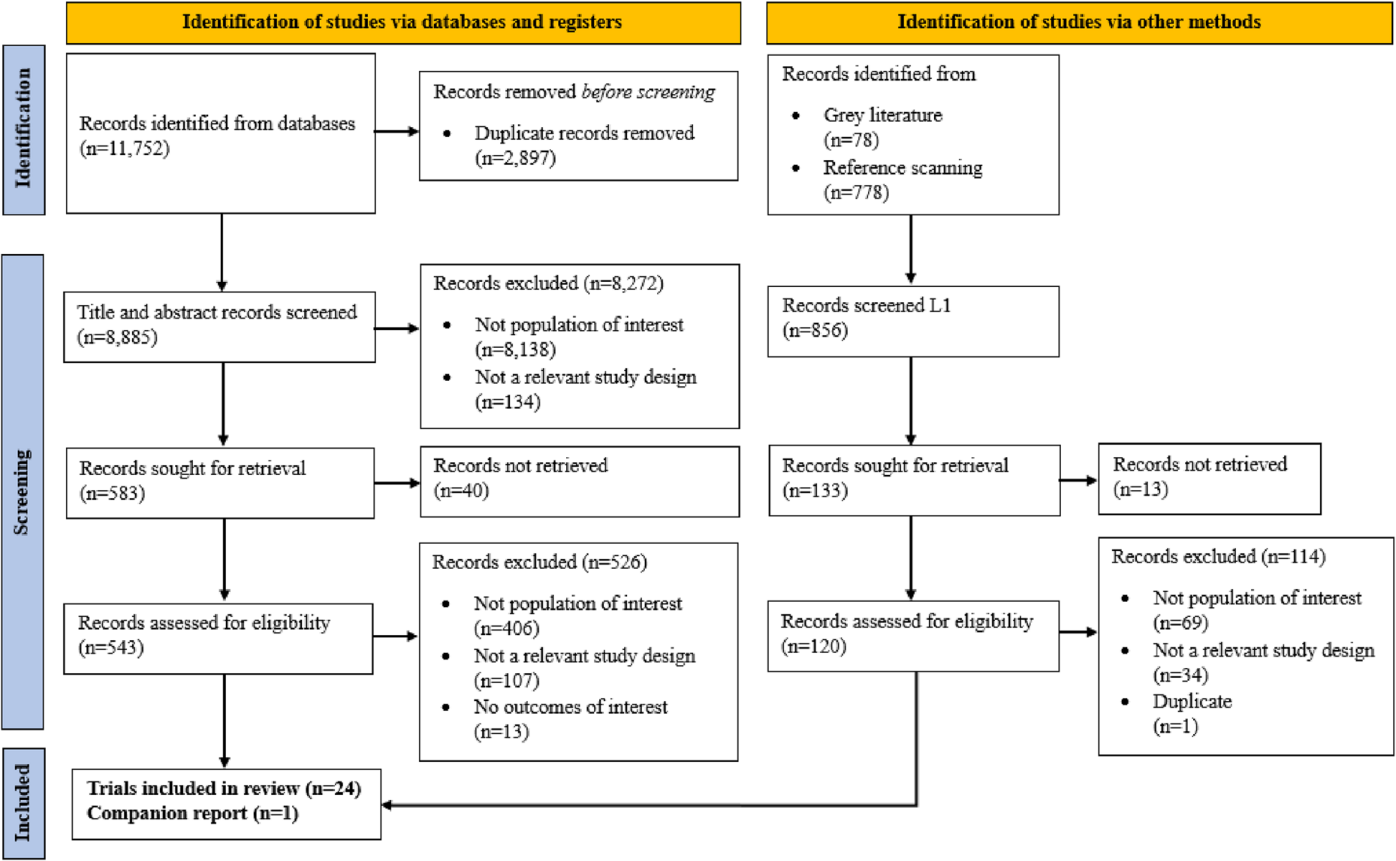
PRISMA flow diagram

After screening 8855 citations from the electronic database searches, 803 extracts from grey literature searches, as well as 663 full-text articles, 24 unique trials [23–45] (including 3 from the grey literature and 3 from included study reference scanning), and 1 companion report [34] (i.e., publications that provided supplementary material to the main trial publication) fulfilled inclusion eligibility criteria (Fig. 1). Brady 2015 included data on two studies which were considered as unique trials [42]. One trial within Huis 2019 was classified as a companion report as it was unclear if the sample was independent from another trial within the same article [34]. All included studies were published in English. A list of studies that were closely related to the inclusion criteria but ultimately excluded is provided in Additional file 1: Appendix 10.

### Study characteristics

Most trials were RCTs randomized at the participant level (*n* = 18) [24–39, 41, 42, 45] and four were randomized at the cluster level [23, 40, 43, 44], while the remaining two were quasi-randomized RCTs [26, 36]. The trials were published between 1979 and 2022, with over 50.0% published since 2017. Trials were predominantly conducted in the USA (*n* = 13). Seventeen of the included trials were conducted in high-income countries (HICs) [23, 24, 26–28, 30–33, 35, 37–39, 41, 42, 45], 2 in middle-income countries (MICs) [25, 43], 4 in lower-middle income countries (LMICs) [29, 34, 40, 44], and one in a combination of LMICs and MICs [36]. Trials were set in workplaces spanning various sectors, including eight in the academic or educational sectors [23, 26–28, 30, 39, 41, 42], five in microfinance [33–35, 43, 44], one in healthcare [45], five in corporate [24, 31, 32, 37, 42], three where the workplace setting was either not clearly described or fictitious [25, 29, 40], one in a military workplace setting [38], and one in a forestry workplace setting [36]. Trials conducted in MICs and LMICs were primarily microfinance initiatives where the focus was on increasing the involvement of women in household finance decisions or expanding their small businesses with their husbands. The setting was multi-site in 12 trials [23, 25, 28, 29, 31, 35, 36, 38, 40, 43–45], single site in 10 trials [24, 26, 27, 30, 33, 34, 37, 39, 42], and 2 trials did not report sufficient information to determine the site setting [32, 41]. Nineteen were directed at the individual level [24–27, 29–31, 33–44], 3 at the organizational level [23, 42, 45], and 2 at both the individual and organizational levels [28, 32]. Further details on the included trials as well as the companion report are available in Additional file 1: Appendix 11.

### Participant characteristics

The total number of participants was 14,798 across all RCTs (Table 1). The median number of participants was 247 across the RCTs, ranging from 23 to 4356 participants (Additional file 1: Appendix 12). The average proportion of participants reported as being females or women was 69.1%. At least one element of the PROGRESS-Plus criteria was reported in 96% of the RCTs (23/24 or 95.8%; Table 1, Additional file 1: Appendix 13). Most RCTs reported the gender/sex (20/24 or 83.3%) and occupation (18/24 or 75.0%) of the included participants. Nine (37.5%) trials reported race/ethnicity, ten (41.7%) reported on education, five on place of residence (20.8%), six on socioeconomic status (25.0%), and three (13.0%) on religion. No RCTs (0%) reported other elements of PROGRESS-Plus, namely, culture, language, social capital, disability, age, features of relationships (e.g., whether or not an individual had children or aging parents under their care), and time-dependent relationships (e.g., new hires, people coming back from a leave of absence, people with time-limited contracts.)

**Table 1:** Summary of study and participant characteristics

Characteristics	Number (%)
Study characteristics (*n* = 24 trials)	
Year of publication	
1979	1 (4.2%)
2007	1 (4.2%)
2012	1 (4.2%)
2015	5 (20.8%)
2017	4 (16.7%)
2018	2 (8.3%)
2019	6 (25.0%)
2020	1 (4.2%)
2021	1 (4.2%)
2022	2 (8.3%)
Geographical region	
Burkina Faso	1 (4.2%)
Japan	1 (4.2%)
Kenya	1 (4.2%)
Norway	1 (4.2%)
Republic of Ireland	1 (4.2%)
Multi-country	1 (4.2%)
Norway	1 (4.2%)
Sri Lanka	1 (4.2%)
Uganda	1 (4.2%)
UK	1 (4.2%)
USA	13 (54.2%)
Vietnam	1 (4.2%)
Study design	
Cluster RCT	4 (16.7%)
Quasi RCT	2 (8.3%)
RCT	18 (75.0%)
Setting	
Multi-site	12 (50.0%)
Single site	10 (41.7%)
NR	2 (8.3%)
Participants characteristics	
Total # participants	14,798
Median number of participants (range)	247 (23.0–4356.0)
Mean % Female- participants(range)	69.1 (13.2–100.0)
Age (mean/median)	
≤ 40 years	6 (25.0%)
> 40 years	5 (20.8%)
Not reported	13 (54.2%)
Studies reporting on PROGRESS items^a^	
Place of residence	5
Race/ethnicity	9
Occupation	18
Gender/sex	20
Religion	3
Education	10
Socioeconomic status	6
Social capital	0

*RCT* randomized control trial, *NR* not reported

^a^Multiple categories reported per study

Most RCTs did not explicitly report a definition of sex or gender (20/24, 83.3%) (Additional file 1: Appendix 14). Most RCTs also used gender terms (i.e., man/woman) interchangeably with sex terms (i.e., male/female, 22/24, 91.7%). Four trials (4/24, 16.7%) provided a definition of sex or gender [33, 35, 39, 40]; of those, one trial provided a definition for both terms. One trial (4.2%) did not conflate sex and gender terminology [35]. Four (16.7%) RCTs reported gender as a variable defined as man/woman [27, 32, 37, 38], and in these cases, it was unclear how this was determined. Five (20.8%) RCTs reported that gender was determined through self-identification via questionnaire [24, 28, 33, 35, 45]. Nine (37.5%) RCTs focused on interventions targeting females or women [23, 25, 34, 41–45]. Two RCTs specifically identified non-binary gender beyond [28] man/woman with categories including “transgender”, “queer/non-binary”, and “other” [35]. All RCTs failed to report proportions of their participants according to their gender identities or roles.

One RCT (4.2%) explored the intersection of gender and race in their analysis reporting—reflected as white men and white women compared to “minority men” and “minority women” [28]. None of the other RCTs reported on intersectionality or the intersection of gender with other variables.

### Intervention characteristics

A pre-existing framework was used to categorize the interventions (Table 2; Additional file 1: Appendices 15–16) into six groupings [46]. The same trial could be categorized into multiple intervention categories. One trial focused on (i) *quantifying gender impacts* by making data (reported by gender) publicly available on work activity in an academic department [28]. Fifteen trials [24, 26–28, 30, 31, 33, 35–40, 42, 44] focused on (ii) *behavioural or systemic changes*, such as recognizing the need for gender equity solutions at the organizational level [27, 28, 36], use of gender-neutral language in recruitment and requests for proposals [30, 42], and use of quotas in terms of number of women and providing training on gender bias [36, 38]. Two trials examined (iii) *career flexibility interventions* [23, 32] such as one trial addressing work–family conflict and another trial examining development of flexible scheduling. Nine trials examined (iv) *increased visibility, recognition and representation interventions*, whereby six studies [23, 25, 34, 41, 43, 44] helped foster careers through interventions to promote manuscript writing in academia and targeted business training in the private sector. In addition, three trials examined leadership programs [28, 29, 45], and one examined role models [41]. Regarding (v) *creating opportunities for development, mentorship and sponsorship interventions,* three trials examined career advising plans [25, 34, 43], and one examined a peer mentoring program [45]. Finally, concerning (vi) *financial support interventions*, four of the included trials focused on microfinance [25, 29, 34, 43, 44]. Of these, three trials focused specifically on females/women [25, 34, 43]. Microfinance studies were included and reported separately as they reported on gender equality and aim to increase gender equality and reduce gender discrimination.

**Table 2:** Summary of intervention outcomes and results

Author, year	(Level of intervention focus) *Intervention category*	Outcome categories addressed	Abstract conclusion
Bapna, 2021 [37]	(Individual)	*(vii) Networking measures*	Positive
Bates, 2019 [33]	(Individual) (ii) *Behavioural or systemic changes*	*(ii) Addressing bias or changes in biases outcome measures* *(iii) Workplace culture outcome measures*	Positive
Brady, 2015 [42] (Two studies are reported in this article)	Study 1: (Organizational); Study 2: (Individual) (ii) *Be*havioural* or systemic changes*	*(iii) Workplace culture outcome measures*	Negative
Bulte, 2017 [25]	(Individual) (iv)* Increased visibility, recognition, and representation interventions* (v)* Creating opportunities for development, mentorship, and sponsorship interventions* (vi) *Financial support interventions*	*(i) Microfinance outcome measures*	Positive
Chinen, 2017 [40]	(Individual) (ii) *Behavioural or systemic changes*	*(ii) Addressing bias or changes in biases outcome measures* *(vi) Education outcome measures*	Neutral
Cook, 2019 [36]	(Individual) (ii) *Behavioural or systemic changes*	*(iv) Gender equity outcome measures*	Positive
Dahl, 2018 [38]	(Individual) (ii) *Behavioural or systemic changes*	*(iv) Gender equity outcome measures*	Positive
Ginter, 2020 [41]	(Individual) (iv)* Increased visibility, recognition, and representation interventions*	(v) A*cademic workforce outcome measures* *(viii) Academic output outcome measures*	Positive
Grisso, 2015 [23]	(Organizational) (iii) *Career flexibility interventions* (iv)* Increased visibility, recognition, and representation interventions*	*(iii) Workplace culture outcome measures* *(viii) Academic output outcome measures*	Positive
Huis, 2019 [34]	(Individual) (iv)* Increased visibility, recognition, and representation interventions* (v)* Creating opportunities for development, mentorship, and sponsorship interventions* (vi) *Financial support interventions*	*(i) Microfinance outcome measures*	Positive
Huis, 2019 [43]	(Individual) (iv)* Increased visibility, recognition, and representation interventions* (v)* Creating opportunities for development, mentorship and sponsorship interventions* (vi*) Financial support interventions*	*(i) Microfinance outcome measures*	Positive
Ismayilova, 2017 [44]	(Individual) (ii) *Behavioural or systemic changes* (iv*) Increased visibility, recognition, and representation interventions* (vi*) Financial support interventions*	*(i) Microfinance outcome measures*	Positive
Matsutaka, 2022 [24]	(Individual) (ii) *Behavioural or systemic changes*	*(ii) Addressing bias or changes in biases outcome measures* *(vi) Education outcome measures*	Positive
O’Meara, 2018 [28]	(Individual/organizational) (i)* Quantifying gender impacts* (ii) *Behavioural or systemic changes* (iv) *Increased visibility, recognition, and representation interventions*	*(iii) Workplace culture outcome measures*	Positive
Paek, 2022 [32]	(Individual/organizational) (iii)* Career flexibility interventions*	*(iii) Workplace culture outcome measures*	Positive
Peterson, 2019 [27]	(Individual) (ii) *Behavioural or systemic changes*	*(v) Academic workforce outcome measures*	Positive
Rivera, 2019 [26]	(Individual) (ii) *Behavioural or systemic changes*	*(ii) Addressing bias or changes in biases outcome measures*	Positive
Shankar, 2015 [29]	(Individual) (iv)* Increased visibility, recognition, and representation interventions* (vi) *Financial support interventions*	*(i) Microfinance outcome measures*	Positive
Smith, 2015 [30]	(Individual) (ii) *Behavioural or systemic changes*	*(iv) Gender equity outcome measures*	Positive
Warren, 2017 [35]	(Individual) (ii) *Behavioural or systemic changes*	*(ii) Addressing bias or changes in biases outcome measures* *(vi) Education outcome measures*	Positive
Webb, 2012 [31]	(Individual)	*(iv) Gender equity outcome measures*	Positive
Wiseman, 1979 [39]	(Individual)	*(iv) Gender equity outcome measures*	Positive
Woolnough, 2007 [45]	(Organizational) (iv) Increased visibility, recognition, and representation interventions (v) Creating opportunities for development, mentorship, and sponsorship interventions	*(iii) Workplace culture outcome measures*	Positive

### Outcome frequencies

Across the 24 included trials, there were 254 outcomes reported (Additional file 1: Appendix 17) that we organized into eight categories: (i) *microfinance outcome measures* were reported 69 times in five (20.8%) trials [25, 29, 34, 43, 44] and typically included measures such as business knowledge, sales and profits totals, goal setting, and self-esteem regarding microfinance interventions. Regarding (ii) *addressing bias or changes in biases outcome measures*, these included use of various scales such as the Neo-sexism scale [33], as well as self-reporting of reductions in implicit homophobia or transphobia [24] biases. Addressing bias or changes in biases outcomes were reported 53 times in five (20.8%) trials [24, 26, 33, 35, 40]. Next, *(iii) workplace culture outcome measures* included changes in work self-efficacy, hours worked per week, and perception of workplace fairness. Workplace culture outcomes were reported 52 times in six (20.8%) trials [23, 28, 32, 42, 45]. Concerning (iv) *gender equity outcome measures*, these included number of women short-listed or interviewed for positions, gender attitudes, and female leadership attitudes. Gender equity outcomes were reported 30 times in 20.8% (5/24) of studies [30, 31, 36, 38, 39]. Outcome category (v) *academic workforce outcome measures* included metrics such as tenure stream jobs, tenured positions, and overall teaching evaluations. Academic workforce outcomes were reported 16 times in 8.3% (2/24) studies [27, 41]. Category (vi) *education outcome measures* included measures pertaining to knowledge and comprehension of the subject matter, as well as increased knowledge of terminology and concepts. Education outcomes were reported 12 times in three (12.5%) trials [24, 35, 40]. Outcome category (vii) *networking measures* included new contacts established and number of LinkedIn connections created after a conference or event. Networking classed outcomes were reported 12 times in 4.2% (1/24) studies [37]. Finally, (viii) *academic output outcome measures* included accruing data about individuals in terms of publications, funding, and other productivity measures. Academic output outcomes were reported 10 times in two (8.3%) of trials [23, 41].

### Conclusion statements (from included studies)

Overall, most conclusion statements according to the abstract “bottom line” were categorized as being positive (21/24, 87.5%), meaning that there was an effect of the intervention. One conclusion statement was categorized as neutral (4.2%) [40] and two as negative (8.3%) [42]. No conclusion statements were presented as being indeterminate.

## Discussion

Our comprehensive scoping review on gender equity interventions in the workplace found that although there may be widespread awareness of issues related to gender (in)equity, that while research interest is building over time, very few intervention studies are examining the gender gap through randomized trials [7], the most methodologically rigorous experimental design. Many of the studies involved a single specific place [38], such as a specific university [23, 30], or a specific conference [37], or questionnaire [27]; and almost all were exclusively held in a specific country. As such, the global reach and scope of gender equity issues has been largely neglected.

In this scoping review, most of the studies come from the USA, yet there is a need for understanding of these issues globally, as workplace culture is not universal across countries. An intervention that is effective in one place may not show the same effectiveness elsewhere. Studies on gender equity in the workplace were conducted sporadically in a handful of other HICs and LMICs. The interventions examined in LMICs focused mostly on getting women more involved in household finance decisions or expanding their small businesses with their husbands. In contrast, the interventions from high-income countries did not focus on the family unit.

A major finding of our scoping review is the lack of standardized methods, outcomes, and definitions in this area and indicate that future research is warranted to standardize this research area. To foster common reporting in this field of gender equity research, we suggest adoption of at least minimal reporting standards around data pertaining to patient characteristics, interventions, and outcomes. We consider definitions of sex and gender to be particularly important, as well as explicit reporting of how sex and gender as variables are determined (i.e., medical reporting, self-reporting), if the variables are only considered as binary characteristics, etc. In terms of minimal reporting standards where equity is concerned, we suggest abiding by the PROGRESS-Plus criteria. Where that is not possible, reporting of education, occupation, race/ethnicity, and economic class, are suggested as bare minimums. Regarding reporting of interventions and outcomes—organization into classes or categories based on previous frameworks is encouraged. Despite the development of tools such as SAGER [18], to support and guide equity reporting, RCTs on gender equity interventions have largely failed to meet these reporting standards. We did not find any improvement over time in reporting.

An additional finding was the lack of rigor associated with sex- or gender-related reporting. Gender, a social variable such as man or woman, was often used interchangeably with the biological variable of sex in the literature we examined. Although definitions were provided in some cases, gender and sex terms were still conflated. Furthermore, just one trial [28] reported on intersectionality in describing their study population by examining gender with race/ethnicity. Other equity variables such as religion, age, or socioeconomic status were variably reported, and not in an intersectional way.

A major strength of this scoping review was the involvement of a public partner on the project who had lived experience with the topic area. By involving this individual, the team contextualized the results using their expertise and experience. According to the GRIPP-2 checklist, facilitators to the engagement need to be discussed. In our review, a facilitator to engagement by the patient partner was the virtual environment in which this research was conducted. A one-page lay summary written by the patient partner can be found in Additional file 1: Appendix 18. According to the GRIPP-2 checklist, amendments to patient partner definitions need to be suggested. Regarding the definition of patient partner that the team used from the Canadian Institutes of Health Research (CIHR), no amendments are suggested, as it is very broad and inclusive. There were no harms mentioned by the patient partner and the experience was positive overall for everyone involved.

### Limitations

We did not appraise the quality or risk of bias in the included studies, which is consistent with the JBI guide for scoping reviews [13]. Although the literature search was broad and not limited to English, we may have missed trials, especially for studies written in languages other than English, which are often not well indexed from specific disciplines (although several discipline-focused databases were searched). We identified few trials from LMIC settings, suggesting the results were more applicable to high income economy contexts. In most trials, only gender identity was considered. There was a lack of consideration for the impacts of gender roles, parental status, or caregiver status. By limiting outcomes to gender identity and not taking other gender (and other intersectional) factors into account, we are unlikely to achieve equity in the future. Protocol deviations include not conducting a living scoping review (i.e., routinely updating the literature search) due to a lack of funding and broadening the focus from academic settings to any workplace setting due to the dearth of literature available.

Most interventions took place in an academic or educational setting, this highlights that the education sector has even further to go to reach gender equity. Due to the heterogeneity of intervention settings, it is important to note that interventions that may work (or not) in one workplace setting may have different outcomes in another workplace setting. This highlights the need to test interventions across multiple workplace and societal settings.

Although many of the conclusion statements were positive, this does not imply that the gender equity interventions work. A future systematic review and meta-analysis would need to confirm these preliminary results. The conclusion statements need to be interpreted with caution, as there is the opportunity to “spin” them in a more favorable way [50] in the abstracts of trials.

While the focus of this review is on formal workplace settings, we would be remiss to not acknowledge that gender inequities are much higher in the informal sector where implementing interventions is difficult [51]. Nearly 60.0% of informal workers are women [52]. We suggest this as an area of focus for future research.

## Conclusions

There is a paucity of scientific literature on interventions to promote workplace gender equity. Few PROGRESS-Plus items were reported. Non-binary gender identities and issues related to intersectionality were not adequately considered. Future research should provide consistent and contemporary definitions of gender and sex, be explicit in how sex or gender is ascertained, and apply sex and gender correctly and appropriately in their correct context. More trials are required examining gender equity interventions in the workplace and future systematic reviews can examine their related effectiveness.

### Supplementary Information

**Additional file 1: Appendix 1.** PRISMA ScR Checklist. **Appendix 2.** SAGER Guidelines. **Appendix 3.** SIITHIA Checklist. **Appendix 4.** GRIPP2 Reporting Checklist. **Appendix 5.** Database Search Strategies. **Appendix 6.** Grey Literature Sources. **Appendix 7.** L1 Screening Form for Titles and Abstracts. **Appendix 8.** L2 Screening Form for Full-Text Articles. **Appendix 9.** Data Abstraction Form. **Appendix 10.** Closely Related but Ultimately Excluded Studies. **Appendix 11.** Participants Characteristics. **Appendix 12.** Participants Characteristics. **Appendix 13.** PROGRESS Plus Table. **Appendix 14.** Definitions of Gender and Sex. **Appendix 15.** Intervention Characteristics. **Appendix 16.** Details of Intervention Outcomes and Results. **Appendix 17.** Outcomes Examined in Included Studies. **Appendix 18.** Patient Partner Lay Summary.

## Data Availability

All of the data are available in Additional file 1.

## Abbreviations

CADTH: Canadian Agency for Drugs and Technologies in Health
CINAHL: Cumulated Index to Nursing and Allied Health Literature
ERIC: Education Resources Information Center Index
GRIPP: Guidance for Reporting Involvement of Patients and the Public
HICs: High-income countries
LMICs: Lower-middle income countries
MICs: Middle-income countries
PAIS Index: Public Affairs Information Service Index
PRESS: Peer Review of Electronic Search Strategies
PROGRESS-Plus equity variables: Place of residence, Race/ethnicity/culture/language, Occupation, Gender/sex, Religion, Education, Socioeconomic status, Social capital
RCTs: Randomized controlled trials
SAGER: Sex and Gender Equity in Research
SIITHIA: Strengthening the Integration of Intersectionality Theory in Health Inequality Analysis

## References

[1] Gender equality still ‘300 years away’, says UN secretary general: The Guardian; 2023. Available from: https://www.theguardian.com/global-development/2023/mar/06/antonio-guterres-un-general-assembly-gender-equality.

[2] Achieve gender equaility and empower women and girls: United Nations; 2023. Available from: https://sdgs.un.org/goals/goal5.

[3] Gender Equality: Bill & Melinda Gates Foundation; 2023. Available from: https://www.gatesfoundation.org/our-work/programs/gender-equality.

[4] Hoffower H, Thier J. Melinda French Gates on her foundation’s shocking findings that gender equality won’t happen for 100 years: ‘Money is power’: Fortune; 2022. Available from: https://fortune.com/2022/09/13/gender-equality-stalled-pandemic-bill-melinda-gates-foundation-study/.

[5] Woetzel J, Madgavkar A, Ellingrud K, Labaye E, Devillard S, Kutcher E (2015). The power of parity: how advancing women's equality can add $12 trillion to global growth.

[6] Women in global health. Policy brief: the state of women and leadership in global health. International: Women in Global Health; 2023.

[7] Tricco AC, Nincic V, Darvesh N, Rios P, Khan PA, Ghassemi MM (2023). Global evidence of gender equity in academic health research: a scoping review. BMJ Open.

[8] Johnson JL, Greaves L, Repta R (2007). Better science with sex and gender: a primer for health research.

[9] PROGRESS-Plus: 2023. Available from: https://methods.cochrane.org/equity/projects/evidence-equity/progress-plus.

[10] Munn Z, Pollock D, Khalil H, Alexander L, Mclnerney P, Godfrey CM (2022). What are scoping reviews? Providing a formal definition of scoping reviews as a type of evidence synthesis. JBI Evid Synth.

[11] Crenshaw K (1989). Demarginalizing the intersection of race and sex: a Black feminist critique of antidiscrimination doctrine, feminist theory and antiracist politics. Univ Chic Leg Forum.

[12] Crenshaw K (1991). Women of color at the center: selections from the third national conference on women of color and the law: mapping the margins: intersectionality, identity politics, and violence against women of color. Stanford Law Rev.

[13] Peters MDJ, Marnie C, Tricco AC, Pollock D, Munn Z, Alexander L (2020). Updated methodological guidance for the conduct of scoping reviews. JBI Evid Synth.

[14] Shamseer L, Moher D, Clarke M, Ghersi D, Liberati A, Petticrew M (2015). Preferred reporting items for systematic review and meta-analysis protocols (PRISMA-P) 2015: elaboration and explanation. BMJ.

[15] Peters MDJ, Godfrey C, McInerney P, Khalil H, Larsen P, Marnie C (2022). Best practice guidance and reporting items for the development of scoping review protocols. JBI Evid Synth.

[16] SPOR Evidence Alliance. Reflective exercise: 2021. Available from: https://sporevidencealliance.ca/wp-content/uploads/2023/02/4.-SPOREA_Reflective-EDI-Exercise-UPDATED_2021.pdf.

[17] Tricco AC, Lillie E, Zarin W, O'Brien KK, Colquhoun H, Levac D (2018). PRISMA extension for scoping reviews (PRISMA-ScR): checklist and explanation. Ann Intern Med.

[18] Heidari S, Babor TF, De Castro P, Tort S, Curno M (2016). Sex and gender equity in research: rationale for the SAGER guidelines and recommended use. Res Integr Peer Rev.

[19] Public Health Agency of Canada. How to integrate intersectionality theory in quantitative health equity analysis? A rapid review and checklist of promising practices: 2022. Available from: https://www.canada.ca/content/dam/phac-aspc/documents/services/publications/science-research-data/how-integrate-intersectionality-theory-quantitative-health-equity-analysis/phac-siithia-checklist.pdf.

[20] Staniszewska S, Brett J, Simera I, Seers K, Mockford C, Goodlad S (2017). GRIPP2 reporting checklists: tools to improve reporting of patient and public involvement in research. BMJ.

[21] McGowan J, Sampson M, Salzwedel DM, Cogo E, Foerster V, Lefebvre C (2016). PRESS Peer Review of Electronic Search Strategies: 2015 Guideline Statement. J Clin Epidemiol.

[22] Canadian Agency for Drugs and Technologies in Health. Grey matters: a tool for searching health-related grey literature. Ottawa: 2023.

[23] Grisso JA, Sammel MD, Rubenstein AH, Speck RM, Conant EF, Scott P (2017). A randomized controlled trial to improve the success of women assistant professors. J Womens Health.

[24] Matsutaka Y, Otsuka Y, Tsuno K, Iida J, Fuji K (2022). Development and evaluation of a training program to reduce homophobia and transphobia among human resource staff and health professionals in the workplace: a randomized controlled trial. Psychol Sex Orientat Gend Divers..

[25] Bulte E, Lensink R, Vu N (2017). Do gender and business trainings affect business outcomes? Experimental evidence from Vietnam. Manag Sci.

[26] Rivera LA, Tilcsik A (2019). Scaling down inequality: rating scales, gender bias, and the architecture of evaluation. Am Sociol Rev.

[27] Peterson DA, Biederman LA, Andersen D, Ditonto TM, Roe K (2019). Mitigating gender bias in student evaluations of teaching. PLoS ONE.

[28] O’Meara K, Jaeger A, Misra J, Lennartz C, Kuvaeva A (2018). Undoing disparities in faculty workloads: a randomized trial experiment. PLoS ONE.

[29] Shankar AV, Onyura M, Alderman J (2015). Agency-based empowerment training enhances sales capacity of female energy entrepreneurs in Kenya. J Health Commun.

[30] Smith JL, Handley IM, Zale AV, Rushing S, Potvin MA (2015). Now hiring! Empirically testing a three-step intervention to increase faculty gender diversity in STEM. Bioscience.

[31] Webb TL, Sheeran P, Pepper J (2012). Gaining control over responses to implicit attitude tests: implementation intentions engender fast responses on attitude-incongruent trials. Br J Soc Psychol.

[32] Paek E (2022). Manager gender and changing attitudes toward schedule control: evidence from the Work, Family, and Health Study. Community Work Fam.

[33] Bates S, Lauve-Moon K, McCloskey R, Anderson-Butcher D (2019). The Gender By Us® Toolkit: a pilot study of an intervention to disrupt implicit gender bias. Affilia.

[34] Huis MA, Hansen N, Otten S, Lensink R (2019). The impact of husbands' involvement in goal-setting training on women's empowerment: first evidence from an intervention among female microfinance borrowers in Sri Lanka. J Community Appl Soc Psychol.

[35] Warren AR (2017). A Video Intervention for Professionals Working with Transgender and Gender Nonconforming Older Adults.

[36] Cook NJ, Grillos T, Andersson KP (2019). Gender quotas increase the equality and effectiveness of climate policy interventions. Nat Clim Chang.

[37] Bapna S, Funk R (2020). Interventions for improving professional networking for women: Experimental evidence from the IT sector. MIS Q.

[38] Dahl GB, Kotsadam A, Rooth D-O (2021). Does integration change gender attitudes? The effect of randomly assigning women to traditionally male teams. Q J Econ.

[39] Wiseman JA (1979). Attitude and behavioral change in academic advisors at Montana State University: sex role stereotyping and sexual bias in vocational choice.

[40] Chinen M, Coombes A, De Hoop T, Castro-Zarzur R, Elmeski M (2017). Can teacher training programs influence gender norms? Mixed-methods experimental evidence from Northern Uganda. J Educ Emerg.

[41] Ginther DK, Currie JM, Blau FD, Croson RT (2020). Can mentoring help female assistant professors in economics? An evaluation by randomized trial. AEA Papers and Proceedings.

[42] Brady LM, Kaiser CR, Major B, Kirby TA (2015). It's fair for us: diversity structures cause women to legitimize discrimination. J Exp Soc Psychol.

[43] Huis M, Lensink R, Vu N, Hansen N (2019). Impacts of the Gender and Entrepreneurship Together Ahead (GET Ahead) training on empowerment of female microfinance borrowers in Northern Vietnam. World Dev.

[44] Ismayilova L, Karimli L, Gaveras E, Tô-Camier A, Sanson J, Chaffin J (2018). An integrated approach to increasing women’s empowerment status and reducing domestic violence: results of a cluster-randomized controlled trial in a West African country. Psychol Violence.

[45] Woolnough HM (2007). A longitudinal study to investigate the impact of a career development and mentoring programme on female mental health nurses.

[46] Tricco AC, Bourgeault I, Moore A, Grunfeld E, Peer N, Straus SE (2021). Advancing gender equity in medicine. CMAJ.

[47] Tricco AC, Straus SE, Moher D (2011). How can we improve the interpretation of systematic reviews?. BMC Med.

[48] Canadian Institutes of Health Research. Glossary of Funding-Related Terms: 2023. Available from: https://cihr-irsc.gc.ca/e/34190.html#p.

[49] SPOR Evidence Alliance. Patient Partner Appreciation Policy and Protocol: 2022. Available from: https://sporevidencealliance.ca/wp-content/uploads/2022/01/SPOREA_Patient-and-Public-Appreciation-Policy_2021.01.14-1.pdf.

[50] Chiu K, Grundy Q, Bero L (2017). ‘Spin’ in published biomedical literature: a methodological systematic review. PLoS Biol.

[51] Women UN (2015). Progress of the world’s women 2015–2016: transforming economies, realizing rights.

[52] International Labour Office (2018). Women and men in the informal economy: a statistical picture.

