# Supplementary material for: Interventions on gender equity in the workplace: a scoping review
Source: BMC Med. 2024 Apr 5;22:149. doi: 10.1186/s12916-024-03346-7 (PMC10998304; doi:10.1186/s12916-024-03346-7)
Supplement: Supplementary file 1 — Additional file 1: Appendix 1. PRISMA ScR Checklist. Appendix 2. SAGER Guidelines. Appendix 3. SIITHIA Checklist. Appendix 4. GRIPP2 Reporting Checklist. Appendix 5. Database Search Strategies. Appendix 6. Grey Literature Sources. Appendix 7. L1 Screening Form for Titles and Abstracts. Appendix 8. L2 Screening Form for Full-Text Articles. Appendix 9. Data Abstraction Form. Appendix 10. Closely Related but Ultimately Excluded Studies. Appendix 11. Participants Characteristics. Appendix 12. Participants Characteristics. Appendix 13. PROGRESS Plus Table. Appendix 14. Definitions of Gender and Sex. Appendix 15. Intervention Characteristics. Appendix 16. Details of Intervention Outcomes and Results. Appendix 17. Outcomes Examined in Included Studies. Appendix 18. Patient Partner Lay Summary. [file 12916_2024_3346_MOESM1_ESM.docx]

Gender Equity: Scoping Review - Appendices

Contents

[Appendix 1 – PRISMA ScR Checklist 2](#_Toc155735662)

[Appendix 2 – SAGER Guidelines 4](#_Toc155735663)

[Appendix 3 – Strengthening the Integration of Intersectionality Theory in Health Inequality Analysis (SIITHIA) checklist 5](#_Toc155735664)

[Appendix 4 – GRIPP2 Reporting Checklist 8](#_Toc155735665)

[Appendix 5 – Database Search Strategies 10](#_Toc155735666)

[Appendix 6 - Grey Literature Sources 21](#_Toc155735667)

[Appendix 7 – L1 Screening Form for Titles and Abstracts 24](#_Toc155735668)

[Appendix 8 – L2 Screening Form for Full-Text Articles 26](#_Toc155735669)

[Appendix 9 – Data Abstraction Form 29](#_Toc155735670)

[Appendix 10 – Closely Related but Ultimately Excluded Studies 39](#_Toc155735671)

[Appendix 11 – Study Characteristics 40](#_Toc155735672)

[Appendix 12 – Participant Characteristics 47](#_Toc155735673)

[Appendix 13 – PROGRESS^1^ Plus Table 51](#_Toc155735674)

[Appendix 14 – Definitions of Gender and Sex 65](#_Toc155735675)

[Appendix 15 – Intervention Characteristics 67](#_Toc155735676)

[Appendix 16 – Details on intervention outcomes and results 72](#_Toc155735677)

[Appendix 17 – Outcomes Examined in Included Studies (*from most to least frequent*) 90](#_Toc155735678)

[Appendix 18 – Patient Partner Lay Summary. 91](#_Toc155735679)

# Appendix 1 – PRISMA ScR Checklist

Preferred Reporting Items for Systematic reviews and Meta-Analyses extension for Scoping Reviews (PRISMA-ScR) Checklist

| **SECTION** | **ITEM** | **PRISMA-ScR CHECKLIST ITEM** | **REPORTED ON PAGE #** |
| --- | --- | --- | --- |
| **TITLE** | | | |
| Title | 1 | Identify the report as a scoping review. | 1 |
| **ABSTRACT** | | | |
| Structured summary | 2 | Provide a structured summary that includes (as applicable): background, objectives, eligibility criteria, sources of evidence, charting methods, results, and conclusions that relate to the review questions and objectives. | 6-7 |
| **INTRODUCTION** | | | |
| Rationale | 3 | Describe the rationale for the review in the context of what is already known. Explain why the review questions/objectives lend themselves to a scoping review approach. | 10 |
| Objectives | 4 | Provide an explicit statement of the questions and objectives being addressed with reference to their key elements (e.g., population or participants, concepts, and context) or other relevant key elements used to conceptualize the review questions and/or objectives. | 11 |
| **METHODS** | | | |
| Protocol and registration | 5 | Indicate whether a review protocol exists; state if and where it can be accessed (e.g., a Web address); and if available, provide registration information, including the registration number. | 11 |
| Eligibility criteria | 6 | Specify characteristics of the sources of evidence used as eligibility criteria (e.g., years considered, language, and publication status), and provide a rationale. | 12-13 |
| Information sources* | 7 | Describe all information sources in the search (e.g., databases with dates of coverage and contact with authors to identify additional sources), as well as the date the most recent search was executed. | 12 |
| Search | 8 | Present the full electronic search strategy for at least 1 database, including any limits used, such that it could be repeated. | Appendix 5 |
| Selection of sources of evidence† | 9 | State the process for selecting sources of evidence (i.e., screening and eligibility) included in the scoping review. | 12-14 |
| Data charting process‡ | 10 | Describe the methods of charting data from the included sources of evidence (e.g., calibrated forms or forms that have been tested by the team before their use, and whether data charting was done independently or in duplicate) and any processes for obtaining and confirming data from investigators. | 13-14 |
| Data items | 11 | List and define all variables for which data were sought and any assumptions and simplifications made. | 12-14, Appendix 7-9 |
| Critical appraisal of individual sources of evidence§ | 12 | If done, provide a rationale for conducting a critical appraisal of included sources of evidence; describe the methods used and how this information was used in any data synthesis (if appropriate). | NA |
| Synthesis of results | 13 | Describe the methods of handling and summarizing the data that were charted. | 14-15 |
| **RESULTS** | | | |
| Selection of sources of evidence | 14 | Give numbers of sources of evidence screened, assessed for eligibility, and included in the review, with reasons for exclusions at each stage, ideally using a flow diagram. | 16, Figure 1 |
| Characteristics of sources of evidence | 15 | For each source of evidence, present characteristics for which data were charted and provide the citations. | 18-23 |
| Critical appraisal within sources of evidence | 16 | If done, present data on critical appraisal of included sources of evidence (see item 12). | NA |
| Results of individual sources of evidence | 17 | For each included source of evidence, present the relevant data that were charted that relate to the review questions and objectives. | Appendix 11-13 |
| Synthesis of results | 18 | Summarize and/or present the charting results as they relate to the review questions and objectives. | Appendix 14-16 |
| **DISCUSSION** | | | |
| Summary of evidence | 19 | Summarize the main results (including an overview of concepts, themes, and types of evidence available), link to the review questions and objectives, and consider the relevance to key groups. | 42-44 |
| Limitations | 20 | Discuss the limitations of the scoping review process. | 44 |
| Conclusions | 21 | Provide a general interpretation of the results with respect to the review questions and objectives, as well as potential implications and/or next steps. | 42-45 |
| **FUNDING** | | | |
| Funding | 22 | Describe sources of funding for the included sources of evidence, as well as sources of funding for the scoping review. Describe the role of the funders of the scoping review. | 46-47 |

JBI = Joanna Briggs Institute; PRISMA-ScR = Preferred Reporting Items for Systematic reviews and Meta-Analyses extension for Scoping Reviews.

* Where *sources of evidence* (see second footnote) are compiled from, such as bibliographic databases, social media platforms, and Web sites.

† A more inclusive/heterogeneous term used to account for the different types of evidence or data sources (e.g., quantitative and/or qualitative research, expert opinion, and policy documents) that may be eligible in a scoping review as opposed to only studies. This is not to be confused with *information sources* (see first footnote).

‡ The frameworks by Arksey and O’Malley (6) and Levac and colleagues (7) and the JBI guidance (4, 5) refer to the process of data extraction in a scoping review as data charting*.*

§ The process of systematically examining research evidence to assess its validity, results, and relevance before using it to inform a decision. This term is used for items 12 and 19 instead of "risk of bias" (which is more applicable to systematic reviews of interventions) to include and acknowledge the various sources of evidence that may be used in a scoping review (e.g., quantitative and/or qualitative research, expert opinion, and policy document).

*From:* Tricco AC, Lillie E, Zarin W, O'Brien KK, Colquhoun H, Levac D, et al. PRISMA Extension for Scoping Reviews (PRISMAScR): Checklist and Explanation. Ann Intern Med. 2018;169:467–473. [doi: 10.7326/M18-0850](http://annals.org/aim/fullarticle/2700389/prisma-extension-scoping-reviews-prisma-scr-checklist-explanation).

# Appendix 2 – SAGER Guidelines

| Recommendations per section of the article | | Page # |
| --- | --- | --- |
| Title and abstract | If only one sex is included in the study, or if the results of the study are to be applied to only one sex or gender, the title and the abstract should specify the sex of animals or any cells, tissues and other material derived from these and the sex and gender of human participants. | NA, all genders included |
| Introduction | Authors should report, where relevant, whether sex and/or gender differences may be expected. | 10-11 |
| Methods | Authors should report how sex and gender were taken into account in the design of the study, whether they ensured adequate representation of males and females, and justify the reasons for any exclusion of males or females. | 11-15 |
| Results | Where appropriate, data should be routinely presented disaggregated by sex and gender. Sex- and gender-based analyses should be reported regardless of positive or negative outcome. In clinical trials, data on withdrawals and dropouts should also be reported disaggregated by sex. | 16-23 |
| Discussion | The potential implications of sex and gender on the study results and analyses should be discussed. If a sex and gender analysis was not conducted, the rationale should be given. Authors should further discuss the implications of the lack of such analysis on the interpretation of the results. | 42-45 |

# Appendix 3 – Strengthening the Integration of Intersectionality Theory in Health Inequality Analysis (SIITHIA) checklist

Public Health Agency of Canada. How to integrate intersectionality theory in quantitative health equity analysis? A rapid review and checklist of promising practices. Ottawa, ON: PHAC; 2022.

| **Study/ Report**  **section** | **Item** | **Promising practice** | | **Page # / Appendix** |
| --- | --- | --- | --- | --- |
| **Introduction** | | | | |
| Background/  Rationale | 1. | Provide a well-referenced definition of intersectionality theory, which  alludes to its central principles* | | 10 |
|  | 2. | Describe inequalities that are consistently observed between population  groups, and that are assumed to be avoidable, as "unjust/unfair" and requiring action. | | 10 |
|  | 3. | Describe the known determinants of the outcome of interest that  operate at, and above, the individual level. | | 10 |
|  | 4. | State and describe underlying assumptions underpinning the study, including a reflexivity* or positionality* statement from the research  team. | | 10 |
|  | 5. | Integrate and summarize evidence developed through research and analysis that involve populations that are affected by the inequalities under study or forms of knowledge that have been under-represented  in public health practice. | | 10 |
| Objectives | 6. | Draw on, and describe literature and complementary theoretical frameworks (including those from outside the field of health sciences),  as needed, to justify and frame the research questions and objectives. | | NA |
|  | 7. | Explore one or more objectives relevant to intersectionality theory,  including: | |  |
|  |  | a) Assessing effect modification or interaction between two or more measures capturing axes of marginalization, in determining health and  social outcomes and inequalities. | | NA |
|  |  | b) Exploring to what extent observed health and social inequalities are explained by a given sub-set of characteristics or factors at the  individual, community, or societal level. | | 14 |
|  |  | c) Assessing changes in determinants, outcomes and inequalities therein (i.e., associations between determinants and outcomes), over  time and across contexts. | | NA |
|  | 8. | Engage with people and populations that are affected by the inequalities under study when establishing research questions and  objectives. | | 15 |
| **Methods** | | | | |
|  | 9. | Engage with populations that are affected by the inequalities under  study, when designing the methods. | | 15 |
| Data source(s) |  | Where possible and relevant to the research question: | |  |
|  | 10. | Collect or use data that allow a comparison of outcomes across  intersecting social positions*. | | 14-16 |
|  | 11. | Collect or use data that allow for an assessment of heterogeneity in determinants and outcomes* across social or spatial units of  aggregation (e.g., schools, regions). | | NA |
|  | 12. | Collect or use data that allow for an assessment of heterogeneity in outcomes across time (including temporal contexts based on calendar  time, and inter-generational and lifecourse perspectives). | NA | |
|  | 13. | Collect or use data that allows for an assessment of independent measures that are hypothetically modifiable, and therefore amenable to  intervention. | NA | |
|  | 14. | Collect or use qualitative data (e.g., using interviews, focus groups, open-ended survey questions, program evaluations, etc.) to complement quantitative data sources, in a mixed-methods research  design approach. | NA | |
| Measures |  | Where relevant to the research question, operationalize independent  measures that enable an assessment of outcomes across: |  | |
|  | 15. | Two or more axes of marginalization; | 14-16, Appendix 11-16 | |
|  | 16. | Units of social or spatial aggregation or clustering; | NA | |
|  | 17. | Temporal contexts (including contexts based on calendar time, and  inter-generational and lifecourse perspectives). | NA | |
|  | 18. | Operationalize and utilize independent measures that are hypothetically  modifiable, and therefore amenable to intervention. | NA | |
|  | 19. | Describe assumptions about the relationship between study measures, including the assumed direction and temporal ordering of associations,  using a causal map or Directed Acyclic Graph*. | NA | |
|  | 20. | Describe assumptions about the broader social phenomena that  measures are assumed to capture or represent. | NA | |
|  | 21. | Describe and justify selected reference categories. | 14-16, Appendix 11-16 | |
| Analysis | 22. | Select/design analyses according to study objectives. For example: |  | |
|  |  | a) For objective 7a (assessing effect modification between two or more measures) potential analyses could include i) stratified analyses, ii) regression-based analyses with interaction terms, iii) or multilevel  analysis of individual heterogeneity and discriminatory accuracy (MAIHDA) analyses | NA | |
|  |  | b) For objective 7b (exploring determinants of inequalities), potential  analyses could include i) causal mediation or ii) decomposition analyses | NA | |
|  |  | c) For objective 7c (assessing changes across time and place), potential analyses could include i) stratified descriptive analyses based on social, temporal, and/or geographic contexts, ii) multivariate mixed-effects  regression analyses | NA | |
|  | 23. | Assess both absolute and relative inequalities between groups. | NA | |
|  | 24. | In regression-based analyses, use a parsimonious set of adjustment  variables based on the causal map described (Item #19). | NA | |
|  | 25. | State and test underlying analytic assumptions using sensitivity  analyses. | NA | |
|  | 26. | Where relevant to the research question, analyze qualitative data, using  methods most appropriate for the study’s objectives. | NA | |
| **Results** | | | | |
|  | 27. | Present and discuss determinants, outcomes, and inequalities therein,  stratified by i) relevant sub-groups, ii) units of space, iii) units of time. | 16-23, Appendix 11-16 | |
|  | 28. | Present and interpret effect modification results, distinguishing  between additive and multiplicative interaction. | NA | |
|  | 29. | If available and relevant for the research question, present the results of the mixed-methods or qualitative analyses that were performed (Item  #26), including key illustrative quotations. | NA | |
| **Discussion** | | | | |
|  | 30. | Engage with populations that are affected by the inequalities under  study, for the review and interpretation of findings. | NA | |
| Findings | 31. | Refer to principles of intersectionality theory when interpreting the  plausible mechanisms explaining results. | 44 | |
| Implications | 32. | Describe the implications of the study for public health practice, as well  as policy and systems change. | 42-45 | |
|  | 33. | Describe the implications of the study for the potential population targets of intervention (e.g., universal policy, targeted/proportional  universalist policy). | 42-45 | |
| Limitations | 34. | Describe how key/core principles of intersectionality were or were not  integrated in the study. | 44-45 | |
|  | 35. | Describe any limitations of data sources (including statistical power),  measures and analyses, and their implications. | 44-45 | |
|  | 36. | Include reflexivity about the power invested in (and reproduced by) the  methods used. | NA | |

# Appendix 4 – GRIPP2 Reporting Checklist

| Section and topic | Item | Reported on page # |
| --- | --- | --- |
| Section 1: Abstract of paper | | |
| 1a Aim | Report the aim of the study | 6 |
| 1b Methods | Describe the methods used by which patients and the public were involved | 6-7 |
| 1c Results | Report the impacts and outcomes of PPI in the study | 7 |
| 1d Conclusions | Summarise the main conclusions of the study | 7 |
| 1e Keywords | Include PPI, “patient and public involvement,” or alternative terms as keywords | NA |
| Section 2: Background to paper | | |
| 2a: Definition | Report the definition of PPI used in the study and how it links to comparable studies | 15 |
| 2b: Theoretical underpinnings | Report the theoretical rationale and any theoretical influences relating to PPI in the study | NA |
| 2c: Concepts and theory development | Report any conceptual or theoretical models, or influences, used in the study | NA |
| Section 3: Aims of paper | | |
| 3: Aim | Report the aim of the study | 11 |
| Section 4: Methods of paper | | |
| 4a: Design | Provide a clear description of methods by which patients and the public were involved | 11-15 |
| 4b: People involved | Provide a description of patients, carers, and the public involved with the PPI activity in the study | 15 |
| 4c: Stages of involvement | Report on how PPI is used at different stages of the study | 15 |
| 4d: Level or nature of involvement | Report the level or nature of PPI used at various stages of the study | 15 |
| Section 5: Capture or measurement of PPI impact | | |
| 5a: Qualitative evidence of impact | If applicable, report the methods used to qualitatively explore the impact of PPI in the study | NA |
| 5b: Quantitative evidence of impact | If applicable, report the methods used to quantitatively measure or assess the impact of PPI | NA |
| 5c: Robustness of measure | If applicable, report the rigour of the method used to capture or measure the impact of PPI | NA |
| Section 6: Economic assessment | | |
| 6: Economic assessment | If applicable, report the method used for an economic assessment of PPI | NA |
| Section 7: Study results | | |
| 7a: Outcomes of PPI | Report the results of PPI in the study, including both positive and negative outcomes | 15 |
| 7b: Impacts of PPI | Report the positive and negative impacts that PPI has had on the research, the individuals involved (including patients and researchers), and wider impacts | 15 |
| 7c: Context of PPI | Report the influence of any contextual factors that enabled or hindered the process or impact of PPI | 15 |
| 7d: Process of PPI | Report the influence of any process factors, that enabled or hindered the impact of PPI | 15 |
| 7ei: Theory development | Report any conceptual or theoretical development in PPI that have emerged | NA |
| 7eii: Theory development | Report evaluation of theoretical models, if any | NA |
| 7f: Measurement | If applicable, report all aspects of instrument development and testing (eg, validity, reliability, feasibility, acceptability, responsiveness, interpretability, appropriateness, precision) | NA |
| 7g: Economic assessment | Report any information on the costs or benefit of PPI | NA |
| Section 8: Discussion and conclusions | | |
| 8a: Outcomes | Comment on how PPI influenced the study overall. Describe positive and negative effects | 43 |
| 8b: Impacts | Comment on the different impacts of PPI identified in this study and how they contribute to new knowledge | 43 |
| 8c: Definition | Comment on the definition of PPI used (reported in the Background section) and whether or not you would suggest any changes | NA |
| 8d: Theoretical underpinnings | Comment on any way your study adds to the theoretical development of PPI | NA |
| 8e: Context | Comment on how context factors influenced PPI in the study | 43 |
| 8f: Process | Comment on how process factors influenced PPI in the study | 43 |
| 8g: Measurement and capture of PPI impact | If applicable, comment on how well PPI impact was evaluated or measured in the study | NA |
| 8h: Economic assessment | If applicable, discuss any aspects of the economic cost or benefit of PPI, particularly any suggestions for future economic modelling. | NA |
| 8i: Reflections/critical perspective | Comment critically on the study, reflecting on the things that went well and those that did not, so that others can learn from this study | 43 |

# Appendix 5 – Database Search Strategies

**Database: Ovid MEDLINE: Epub Ahead of Print, In-Process & Other Non-Indexed Citations, Ovid MEDLINE® Daily and Ovid MEDLINE® <1946-Present>**

1 exp Gender Identity/

2 gender difference?.mp.

3 (sex disparit* or sex difference?).mp.

4 gender identit*.mp.

5 sex role?.mp.

6 (wom#n* adj2 role?).mp.

7 ((man* adj2 role?) or (men* adj2 role?)).mp.

8 gender* role?.mp.

9 (gender* adj3 (analys#s or authorship? or balanc* or bias* or capacity-building or characteristic? or comparison? or composition? or difference? or discrepanc* or discriminat* or disparit* or distribut* or divers* or equal* or equit* or exclusi* or gap or gaps or harass* or imbalanc* or inclusi* or inequalit* or in-equalit* or inequit* or in-equit* or issue or issues or objectif* or parity or prejudic* or proportion* or represent* or status* or stereotyp* or stereo-typ* or stigma* or structure? or trend* or underrepresent* or under-represent* or unequal* or un-equal* or variation?)).tw,kf.

10 ((female? or wom#n* or male or males or man or "man's" or men or "men's" or feminin* or feminis* or masculin* or sex or sexual) adj3 (balanc* or bias* or capacity-building or discrepanc* or discriminat* or disparit* or equal* or equit* or gap or gaps or harass* or identit* or imbalanc* or inclusi* or inequalit* or in-equal* or inequit* or inequit* or objectif* or parity or prejudic* or represent* or status* or stereotyp* or stereo-typ* or stigma* or underrepresent* or under-represent* or unequal* or un-equal*)).tw,kf.

11 ((male? or masculin* or man or men or female? or feminin* or feminis* or wom#n or sex or sexes or sexual) adj3 ((nontraditional* or non-traditional*) adj4 role*)).tw,kf.

12 (femaleness or maleness).tw,kf.

13 Sexism/

14 (sexism* or sexist?).tw,kf.

15 (androcentris* or andro-centris* or patriarch*).tw,kf.

16 ((abhor* or animosit* or antipath* or avers* or dislik* or distast* or grudg* or hate or hates or hated or hating or hatred or ill feeling? or ill will) adj3 (female? or wom#n)).tw,kf.

17 (hostil* adj3 (female? or wom#n)).tw,kf.

18 (hostil* adj3 (man or men or male?)).tw,kf.

19 misogyn*.tw,kf.

20 glass ceiling?.tw,kf.

21 Transgender Persons/

22 (transgender* or trans-gender* or transexual* or tran-sexual* or transsexual* or trans-sexual*).tw,kf.

23 (transmale? or trans male? or trans man or trans men).tw,kf.

24 (transfemale? or trans female? or trans wom#n*).tw,kf.

25 (two spirit$2 or "2 spirit$2").tw,kf.

26 (nonbinary or non-binary).tw,kf.

27 (cisgender* or cis gender*).tw,kf.

28 (gender? adj2 fluid*).tw,kf.

29 (intersectional* or inter-sectional*).tw,kf.

30 ((intersect* or inter-sect*) adj2 (categor* or lens*)).tw,kf.

31 or/1-30 [GENDER FILTER]

32 Sexism/pc [prevention & control]

33 ((sexism* or sexist?) adj3 (abolish* or chang* or control* or counter* or discourag* or eliminat* or free* or mitigat* or policy or policies or prevent* or reduc* or reform* or remov* or shift* or stop*)).tw,kf.

34 Sexual Harassment/pc [prevention & control]

35 (harass* adj3 (abolish* or chang* or control* or counter* or discourag* or eliminat* or free* or mitigat* or policy or policies or prevent* or reduc* or reform* or remov* or shift* or stop*)).tw,kf.

36 Prejudice/pc [prevention & control]

37 ((bias* or discriminat* or inequal* or inequit* or prejudic* or stereotyp* or stereo-typ*) adj3 (abolish* or chang* or control* or counter* or discourag* or eliminat* or free* or mitigat* or policy or policies or prevent* or reform* or reduc* or remov* or shift* or stop*)).tw,kf.

38 ((diversit* or equal* or equit* or parit*) adj3 (advoca* or affirm* or balanc* or champion* or encourag* or facilitat* or favo?r* or incenti* or inspir* or mobilis* or mobiliz* or promot* or reform* or stimulat* or support*)).tw,kf.

39 Cultural Diversity/

40 Quality Improvement/

41 (quality adj3 (advanc* or ameliorat* or better* or boost* or champion* or enhanc* or improv* or optim* or promot* or rais* or refin* or reform*)).tw,kf.

42 (aware* adj3 (advanc* or ameliorat* or better* or boost* or champion* or enhanc* or improv* or optim* or promot* or rais* or refin* or reform*)).tw,kf.

43 ((alter* or chang* or curb* or reform* or shift* or transform*) adj3 (attitud* or belief? or habit*)).tw,kf.

44 Mentors/

45 mentor*.tw,kf.

46 Personnel Selection/

47 (personnel adj3 (hire? or hiring or policy or policies or procedur* or recruit* or reform* or select*)).tw,kf.

48 ((hire? or hiring) adj3 (policy or policies or procedur* or reform*)).tw,kf.

49 exp Organizational Innovation/

50 ((academ* or business* or corporat* or education* or facult* or government* or institution* or law or legal or organi#ation* or universit*) adj3 innovat*).tw,kf.

51 Organizational Policy/

52 ((academ* or business* or corporat* or education* or facult* or government* or institution* or law or legal or organi#ation* or universit*) adj3 (goal? or mandate* or policy or policies or reform* or strateg*)).tw,kf.

53 Women's Rights/

54 ((wom#n* or man or men or "man's" or "men's") adj2 rights).tw,kf.

55 (gender* adj3 (awareness* or program* or train* or workshop*)).tw,kf.

56 ombuds*.tw,kf.

57 affirmative action?.tw,kf.

58 (equal adj3 (access or opportunit*)).tw,kf.

59 egalitarian*.tw,kf.

60 meritocra*.tw,kf.

61 intervention*.ti,kf.

62 initiative*.ti,kf.

63 innovation*.ti,kf.

64 or/32-63 [INTERVENTIONS - GENERAL]

65 31 and 64 [GENDER EQUITY - INTERVENTIONS - GENERAL]

66 exp Animals/ not Humans/

67 65 not 66 [ANIMAL-ONLY REMOVED]

68 (comment or editorial or news or newspaper article).pt.

69 67 not 68 [OPINION PIECES REMOVED]

70 systematic review.pt.

71 exp systematic reviews as topic/

72 meta analysis.pt.

73 exp meta-analysis as topic/

74 (meta-analy* or metanaly* or metaanaly* or met analy* or integrative research or integrative review* or integrative overview* or research integration or research overview* or collaborative review*).tw,kf.

75 (systematic review* or systematic overview* or evidence-based review* or evidence-based overview* or (evidence adj3 (review* or overview*)) or meta-review* or meta-overview* or meta-synthes* or rapid review* or "review of reviews" or umbrella review? or technology assessment* or HTA or HTAs).tw,kf.

76 exp Technology assessment, biomedical/

77 (cochrane or health technology assessment or evidence report or systematic reviews).jw.

78 (network adj (MA or MAs)).tw,kf.

79 (NMA or NMAs or MTC or MTCs or MAIC or MAICs).tw,kf.

80 indirect* compar*.tw,kf.

81 (indirect treatment* adj1 compar*).tw,kf.

82 (mixed treatment* adj1 compar*).tw,kf.

83 (multiple treatment* adj1 compar*).tw,kf.

84 (multi-treatment* adj1 compar*).tw,kf.

85 simultaneous* compar*.tw,kf.

86 mixed comparison?.tw,kf.

87 or/70-86 [REVIEW FILTER]

88 69 and 87 [GENDER EQUITY - REVIEWS]

89 (controlled clinical trial or randomized controlled trial or pragmatic clinical trial or equivalence trial).pt.

90 clinical trials as topic/

91 exp Randomized Controlled Trials as Topic/

92 (randomi#ed or randomi#ation? or randomly or RCT or placebo*).tw,kf.

93 ((singl* or doubl* or trebl* or tripl*) adj (mask* or blind* or dumm*)).tw,kf.

94 trial.ti.

95 or/89-94 [RCT FILTER]

96 69 and 95 [GENDER EQUITY - RCTs]

97 88 or 96 [GENDER EQUITY - REVIEWS/RCTs]

98 Controlled Clinical Trials as Topic/

99 (control* adj2 trial).tw,kf.

100 Non-Randomized Controlled Trials as Topic/

101 (nonrandom* or non-random* or quasi-random* or quasi-experiment*).tw,kf.

102 (nRCT or nonRCT or non-RCT).tw,kf.

103 Controlled Before-After Studies/

104 (control* adj3 ("before and after" or "before after")).tw,kf.

105 Interrupted Time Series Analysis/

106 time series.tw,kf.

107 (pre- adj3 post-).tw,kf.

108 (pretest adj3 posttest).tw,kf.

109 Historically Controlled Study/

110 (control* adj2 study).tw,kf.

111 Control Groups/

112 (control* adj2 group?).tw,kf.

113 or/98-112 [NON-RCT FILTER]

114 69 and 113 [GENDER EQUITY - NON-RCTS]

115 97 or 114 [GENDER EQUITY - REVIEWS/RCTs/nRCTs]

116 exp Cohort Studies/

117 cohort?.tw,kf.

118 Retrospective Studies/

119 (longitudinal or prospective or retrospective).tw,kf.

120 ((followup or follow-up) adj (study or studies)).tw,kf.

121 Observational study.pt.

122 (observation$2 adj (study or studies)).tw,kf.

123 ((population or population-based) adj (study or studies or analys#s)).tw,kf.

124 ((multidimensional or multi-dimensional) adj (study or studies)).tw,kf.

125 Comparative Study.pt.

126 ((comparative or comparison) adj (study or studies)).tw,kf.

127 exp Case-Control Studies/

128 ((case-control* or case-based or case-comparison) adj (study or studies)).tw,kf.

129 or/116-128 [OBSERVATIONAL STUDY FILTER]

130 69 and 129 [GENDER EQUITY - OBSERVATIONAL STUDIES]

131 115 or 130 [GENDER EQUITY - REVIEWS/RCTs/nRCTs/OBSERVATIONAL STUDIES]

**Database: Embase Classic+Embase** <1947 to 2020 January 30>

1 exp gender identity/

2 gender differences.mp.

3 (sex disparit* or sex difference?).mp.

4 gender identit*.mp.

5 sex role/

6 sex role?.mp.

7 (wom#n* adj2 role?).mp.

8 ((man* adj2 role?) or (men* adj2 role?)).mp.

9 gender* role?.mp.

10 (gender* adj3 (analys#s or authorship? or balanc* or bias* or capacity-building or characteristic? or comparison? or composition? or difference? or discrepanc* or discriminat* or disparit* or distribut* or divers* or equal* or equit* or exclusi* or gap or gaps or harass* or imbalanc* or inclusi* or inequalit* or in-equalit* or inequit* or in-equit* or issue or issues or objectif* or parity or prejudic* or proportion* or represent* or status* or stereotyp* or stereo-typ* or stigma* or structure? or trend* or underrepresent* or under-represent* or unequal* or un-equal* or variation?)).tw,kw.

11 ((female? or wom#n* or male or males or man or "man's" or men or "men's" or feminin* or feminis* or masculin* or sex or sexual) adj3 (balanc* or bias* or capacity-building or discrepanc* or discriminat* or disparit* or equal* or equit* or gap or gaps or harass* or identit* or imbalanc* or inclusi* or inequalit* or in-equal* or inequit* or inequit* or objectif* or parity or prejudic* or represent* or status* or stereotyp* or stereo-typ* or stigma* or underrepresent* or under-represent* or unequal* or un-equal*)).tw,kw.

12 ((male? or masculin* or man or men or female? or feminin* or feminis* or wom#n or sex or sexes or sexual) adj3 ((nontraditional* or non-traditional*) adj4 role*)).tw,kw.

13 (femaleness or maleness).tw,kw.

14 exp sexism/

15 (sexism* or sexist?).tw,kw.

16 (androcentris* or andro-centris* or patriarch*).tw,kw.

17 ((abhor* or animosit* or antipath* or avers* or dislik* or distast* or grudg* or hate or hates or hated or hating or hatred or ill feeling? or ill will) adj3 (female? or wom#n)).tw,kw.

18 (hostil* adj3 (female? or wom#n)).tw,kw.

19 (hostil* adj3 (man or men or male?)).tw,kw.

20 misogyn*.tw,kw.

21 glass ceiling?.tw,kw.

22 exp transgender/

23 (transgender* or trans-gender* or transexual* or tran-sexual* or transsexual* or trans-sexual*).tw,kw.

24 (transmale? or trans male? or trans man or trans men).tw,kw.

25 (transfemale? or trans female? or trans wom#n*).tw,kw.

26 (two spirit$2 or "2 spirit$2").tw,kw.

27 (nonbinary or non-binary).tw,kw.

28 (cisgender* or cis gender*).tw,kw.

29 (gender? adj2 fluid*).tw,kw.

30 (intersectional* or inter-sectional*).tw,kw.

31 [((intersect* or inter-sect*) adj2 (categor* or lens*)).tw,kf.]

32 or/1-31 [GENDER FILTER]

33 exp sexism/pc [prevention & control]

34 ((sexism* or sexist?) adj3 (abolish* or chang* or control* or counter* or discourag* or eliminat* or free* or mitigat* or policy or policies or prevent* or reduc* or reform* or remov* or shift* or stop*)).tw,kw.

35 exp sexual harassment/pc [Prevention]

36 (harass* adj3 (abolish* or chang* or control* or counter* or discourag* or eliminat* or free* or mitigat* or policy or policies or prevent* or reduc* or reform* or remov* or shift* or stop*)).tw,kw.

37 prejudice/pc [Prevention]

38 ((bias* or discriminat* or inequal* or inequit* or prejudic* or stereotyp* or stereo-typ*) adj3 (abolish* or chang* or control* or counter* or discourag* or eliminat* or free* or mitigat* or policy or policies or prevent* or reform* or reduc* or remov* or shift* or stop*)).tw,kw.

39 ((diversit* or equal* or equit* or parit*) adj3 (advoca* or affirm* or balanc* or champion* or encourag* or facilitat* or favo?r* or incenti* or inspir* or mobilis* or mobiliz* or promot* or reform* or stimulat* or support*)).tw,kw.

40 cultural diversity/

41 total quality management/

42 (quality adj3 (advanc* or ameliorat* or better* or boost* or champion* or enhanc* or improv* or optim* or promot* or rais* or refin* or reform*)).tw,kw.

43 (aware* adj3 (advanc* or ameliorat* or better* or boost* or champion* or enhanc* or improv* or optim* or promot* or rais* or refin* or reform*)).tw,kw.

44 ((alter* or chang* or curb* or reform* or shift* or transform*) adj3 (attitud* or belief? or habit*)).tw,kw.

45 mentor/

46 mentor*.tw,kw.

47 personnel management/ or health care personnel management/ or hospital personnel management/

48 (personnel adj3 (hire? or hiring or policy or policies or procedur* or recruit* or reform* or select*)).tw,kw.

49 ((hire? or hiring) adj3 (policy or policies or procedur* or reform*)).tw,kw.

50 ((academ* or business* or corporat* or education* or facult* or government* or institution* or law or legal or organi#ation* or universit*) adj3 innovat*).tw,kw.

51 organizational policy/

52 ((academ* or business* or corporat* or education* or facult* or government* or institution* or law or legal or organi#ation* or universit*) adj3 (goal? or mandate* or policy or policies or reform* or strateg*)).tw,kw.

53 women's rights/

54 ((wom#n* or man or men or "man's" or "men's") adj2 rights).tw,kw.

55 (gender* adj3 (awareness* or program* or train* or workshop*)).tw,kw.

56 ombuds*.tw,kw.

57 affirmative action/

58 affirmative action?.tw,kw.

59 (equal adj3 (access or opportunit*)).tw,kw.

60 egalitarian*.tw,kw.

61 meritocra*.tw,kw.

62 intervention*.ti,kw.

63 initiative*.ti,kw.

64 innovation*.ti,kw.

65 or/33-64 [INTERVENTIONS - GENERAL]

66 32 and 65 [GENDER IDENTIFY - INTERVENTIONS - GENERAL]

67 exp animal/ or exp animal experimentation/ or exp animal model/ or exp animal experiment/ or nonhuman/ or exp vertebrate/

68 exp human/ or exp human experimentation/ or exp human experiment/

69 67 not 68

70 66 not 69 [ANIMAL-ONLY REMOVED]

71 editorial.pt.

72 letter.pt. not (letter.pt. and randomized controlled trial/)

73 70 not (71 or 72) [OPINION PIECES REMOVED]

74 meta-analysis/

75 "systematic review"/

76 "meta analysis (topic)"/

77 "systematic review (topic)"/

78 (meta-analy* or metanaly* or metaanaly* or met analy* or integrative research or integrative review* or integrative overview* or research integration or research overview* or collaborative review*).tw,kw.

79 (systematic review* or systematic overview* or evidence-based review* or evidence-based overview* or (evidence adj3 (review* or overview*)) or meta-review* or meta-overview* or meta-synthes* or "review of reviews" or umbrella review? or technology assessment* or HTA or HTAs).tw,kw.

80 biomedical technology assessment/

81 (cochrane or health technology assessment or evidence report).jw.

82 (network adj (MA or MAs)).tw,kw.

83 (NMA or NMAs or MTC or MTCs or MAIC or MAICs).kw,tw. 84 indirect* compar*.tw,kw.

85 (indirect treatment* adj1 compar*).tw,kw.

86 (mixed treatment* adj1 compar*).tw,kw.

87 (multiple treatment* adj1 compar*).tw,kw.

88 (multi-treatment* adj1 compar*).tw,kw.

89 simultaneous* compar*.tw,kw.

90 mixed comparison?.tw,kw.

91 or/74-90 [REVIEW FILTER]

92 73 and 91 [REVIEWS]

93 exp randomized controlled trial/ or controlled clinical trial/

94 "clinical trial (topic)"/ or exp "controlled clinical trial (topic)"/

95 (randomi#ed or randomi#ation? or randomly or RCT or placebo*).tw,kw.

96 ((singl* or doubl* or trebl* or tripl*) adj (mask* or blind* or dumm*)).tw,kw.

97 trial.ti.

98 or/93-97 [RCT FILTER]

99 73 and 98 [RCTs]

100 controlled clinical trial/

101 "controlled clinical trial (topic)"/

102 (control* adj2 trial).tw,kw.

103 (nonrandom* or non-random* or quasi-random* or quasi-experiment*).tw,kw.

104 (nRCT or nonRCT or non-RCT).tw,kw.

105 (control* adj3 ("before and after" or "before after")).tw,kw.

106 time series analysis/

107 time series.tw,kw.

108 pretest posttest control group design/

109 (pre- adj3 post-).tw,kw.

110 (pretest adj3 posttest).tw,kw.

111 controlled study/

112 (control* adj2 study).tw,kw.

113 control group/

114 (control* adj2 group?).tw,kw.

115 or/100-114 [nRCT FILTER]

116 73 and 115 [nRCTs]

117 cohort analysis/

118 cohort?.tw,kw.

119 retrospective study/

120 longitudinal study/

121 prospective study/

122 (longitudinal or prospective or retrospective).tw,kw.

123 follow up/

124 ((followup or follow-up) adj (study or studies)).tw,kw.

125 observational study/

126 (observation$2 adj (study or studies)).tw,kw.

127 population research/

128 ((population or population-based) adj (study or studies or analys#s)).tw,kw.

129 ((multidimensional or multi-dimensional) adj (study or studies)).tw,kw.

130 exp comparative study/

131 ((comparative or comparison) adj (study or studies)).tw,kw.

132 exp case control study/

133 ((case-control* or case-based or case-comparison) adj (study or studies)).tw,kw.

134 or/117-133 [OBSERVATIONAL FILTER]

135 73 and 134 [OBSERVATIONAL STUDIES]

136 92 or 99 or 116 or 135 [ALL STUDY DESIGNS]

**Database: PsycINFO <1806 to January Week 4 2020>**

1 exp Gender Identity/

2 Gender Equality/

3 Gender Gap/

4 "Transgender (Attitudes Toward)"/

5 gender difference?.mp.

6 (sex disparit* or sex difference?).mp.

7 gender identity.mp.

8 Sex Roles/

9 exp "Sex Role Attitudes"/

10 sex role?.mp.

11 (wom#n* adj2 role?).mp.

12 ((man* adj2 role?) or (men* adj2 role?)).mp.

13 gender* role?.mp.

14 (gender* adj3 (analys#s or authorship? or balanc* or bias* or capacity-building or characteristic? or comparison? or composition? or difference? or discrepanc* or discriminat* or disparit* or distribut* or divers* or equal* or equit* or exclusi* or gap or gaps or harass* or imbalanc* or inclusi* or inequalit* or in-equalit* or inequit* or in-equit* or issue or issues or objectif* or parity or prejudic* or proportion* or represent* or status* or stereotyp* or stereo-typ* or stigma* or structure? or trend* or underrepresent* or under-represent* or unequal* or un-equal* or variation?)).tw.

15 Sex Discrimination/

16 ((female? or wom#n* or male or males or man or "man's" or men or "men's" or feminin* or feminis* or masculin* or sex or sexual) adj3 (balanc* or bias* or capacity-building or discrepanc* or discriminat* or disparit* or equal* or equit* or gap or gaps or harass* or identit* or imbalanc* or inclusi* or inequalit* or in-equal* or inequit* or inequit* or objectif* or parity or prejudic* or represent* or status* or stereotyp* or stereo-typ* or stigma* or underrepresent* or under-represent* or unequal* or un-equal*)).tw.

17 ((male? or masculin* or man or men or female? or feminin* or feminis* or wom#n or sex or sexes or sexual) adj3 ((nontraditional* or non-traditional*) adj4 role*)).tw.

18 (femaleness or maleness).tw.

19 Sexism/

20 (sexism* or sexist?).tw.

21 (androcentris* or andro-centris* or patriarch*).tw.

22 ((abhor* or animosit* or antipath* or avers* or dislik* or distast* or grudg* or hate or hates or hated or hating or hatred or ill feeling? or ill will) adj3 (female? or wom#n)).tw.

23 (hostil* adj3 (female? or wom#n)).tw.

24 (hostil* adj3 (man or men or male?)).tw.

25 misogyn*.tw.

26 glass ceiling?.tw.

27 (transgender* or trans-gender* or transexual* or tran-sexual* or transsexual* or trans-sexual*).tw.

28 (transmale? or trans male? or trans man or trans men).tw.

29 (transfemale? or trans female? or trans wom#n*).tw.

30 (two spirit$2 or "2 spirit$2").tw.

31 (nonbinary or non-binary).tw.

32 (cisgender* or cis gender*).tw.

33 (gender? adj2 fluid*).tw.

34 (intersectional* or inter-sectional*).tw.

35 ((intersect* or inter-sect*) adj2 (categor* or lens*)).tw.

36 or/1-35 [GENDER FILTER]

37 ((sexism* or sexist?) adj3 (abolish* or chang* or control* or counter* or discourag* or eliminat* or free* or mitigat* or policy or policies or prevent* or reduc* or reform* or remov* or shift* or stop*)).tw.

38 (harass* adj3 (abolish* or chang* or control* or counter* or discourag* or eliminat* or free* or mitigat* or policy or policies or prevent* or reduc* or reform* or remov* or shift* or stop*)).tw.

39 ((bias* or discriminat* or inequal* or inequit* or prejudic* or stereotyp* or stereo-typ*) adj3 (abolish* or chang* or control* or counter* or discourag* or eliminat* or free* or mitigat* or policy or policies or prevent* or reform* or reduc* or remov* or shift* or stop*)).tw.

40 ((diversit* or equal* or equit* or parit*) adj3 (advoca* or affirm* or balanc* or champion* or encourag* or facilitat* or favo?r* or incenti* or inspir* or mobilis* or mobiliz* or promot* or reform* or stimulat* or support*)).tw.

41 diversity in the workplace/

42 cultural sensitivity/

43 (quality adj3 (advanc* or ameliorat* or better* or boost* or champion* or enhanc* or improv* or optim* or promot* or rais* or refin* or reform*)).tw.

44 (aware* adj3 (advanc* or ameliorat* or better* or boost* or champion* or enhanc* or improv* or optim* or promot* or rais* or refin* or reform*)).tw.

45 ((alter* or chang* or curb* or reform* or shift* or transform*) adj3 (attitud* or belief? or habit*)).tw.

46 mentor/

47 mentor*.tw.

48 personnel selection/

49 (personnel adj3 (hire? or hiring or policy or policies or procedur* or recruit* or reform* or select*)).tw.

50 ((hire? or hiring) adj3 (policy or policies or procedur* or reform*)).tw.

51 ((academ* or business* or corporat* or education* or facult* or government* or institution* or law or legal or organi#ation* or universit*) adj3 innovat*).tw.

52 ((academ* or business* or corporat* or education* or facult* or government* or institution* or law or legal or organi#ation* or universit*) adj3 (goal? or mandate* or policy or policies or reform* or strateg*)).tw.

53 ((wom#n* or man or men or "man's" or "men's") adj2 rights).tw.

54 (gender* adj3 (awareness* or program* or train* or workshop*)).tw.

55 ombuds*.tw.

56 affirmative action/

57 affirmative action?.tw.

58 (equal adj3 (access or opportunit*)).tw.

59 egalitarianism/

60 egalitarian*.tw.

61 meritocra*.tw.

62 workplace intervention/

63 intervention*.ti.

64 initiative*.ti.

65 innovation/

66 innovation.ti.

67 or/37-66 [INTERVENTIONS - GENERAL]

68 36 and 67 [GENDER EQUITY - INTERVENTIONS - GENERAL]

69 "systematic review"/

70 meta analysis/

71 (meta-analy* or metanaly* or metaanaly* or met analy* or integrative research or integrative review* or integrative overview* or research integration or research overview* or collaborative review*).tw.

72 (systematic review* or systematic overview* or evidence-based review* or evidence-based overview* or (evidence adj3 (review* or overview*)) or meta-review* or meta-overview* or meta-synthes* or rapid review* or "review of reviews" or umbrella review? or technology assessment* or HTA or HTAs).tw.

73 (network adj (MA or MAs)).tw.

74 (NMA or NMAs or MTC or MTCs or MAIC or MAICs).tw.

75 indirect* compar*.tw.

76 (indirect treatment* adj1 compar*).tw.

77 (mixed treatment* adj1 compar*).tw.

78 (multiple treatment* adj1 compar*).tw.

79 (multi-treatment* adj1 compar*).tw.

80 simultaneous* compar*.tw.

81 mixed comparison?.tw.

82 or/69-81 [REVIEW FILTER]

83 68 and 82 [REVIEWS]

84 exp randomized controlled trials/

85 (randomi#ed or randomi#ation? or randomly or RCT or placebo*).tw.

86 ((singl* or doubl* or trebl* or tripl*) adj (mask* or blind* or dumm*)).tw.

87 trial.ti.

88 or/84-87 [RCT FILTER]

89 68 and 88 [RCTs]

90 clinical trials/

91 (nonrandom* or non-random* or quasi-random* or quasi-experiment*).tw.

92 (nRCT or nonRCT or non-RCT).tw.

93 (control* adj3 ("before and after" or "before after")).tw.

94 time series/

95 time series.tw.

96 Pretesting/ and Posttesting/

97 (pre- adj3 post-).tw.

98 (pretest adj3 posttest).tw.

99 (control* adj2 study).tw.

100 experiment controls/

101 (control* adj2 group?).tw.

102 or/90-101 [nRCT FILTER]

103 68 and 102 [nRCTs]

104 cohort?.tw.

105 retrospective studies/

106 longitudinal studies/

107 prospective studies/

108 (longitudinal or prospective or retrospective).tw.

109 followup studies/

110 ((followup or follow-up) adj (study or studies)).tw.

111 (observation$2 adj (study or studies)).tw.

112 ((population or population-based) adj (study or studies or analys#s)).tw.

113 ((multidimensional or multi-dimensional) adj (study or studies)).tw.

114 ((comparative or comparison) adj (study or studies)).tw.

115 ((case-control* or case-based or case-comparison) adj (study or studies)).tw.

116 or/104-115 [OBSERVATIONAL STUDY FILTER]

117 68 and 116 [OBSERVATIONAL STUDIES]

118 83 or 89 or 103 or 117 [ALL STUDY DESIGNS]

# Appendix 6 - Grey Literature Sources

Academic Women's Association: University of Alberta:

https://uofaawa.wordpress.com/awadiversity-gap-campaign/

ADVANCE: Increasing the Participation and Advancement of Women in Academic Science

and Engineering Careers: [https://www.colorado.edu/eer/research-areas/women-science](about:blank)

American Medical Women’s Association: [https://www.amwa-doc.org/our-work/initiatives/gender-equity-task-force/](about:blank)

CCGSD – Canadian Centre for Gender & Sexual Diversity: [http://ccgsd-ccdgs.org](about:blank)

Centre for Gender Advocacy - Fight gender oppression!: [www.genderadvocacy.org](about:blank)

CIHI: [https://www.cihi.ca/](about:blank)

CIHR Gender and Health Institute: [http://www.cihr-irsc.gc.ca/e/8681.html](about:blank)

European Gender Portal for Equality in Science: [http://www.genderportal.eu/](about:blank)

Humboldt Foundation: [https://www.humboldt-foundation.de/web/home.html](about:blank)

Institute of International Education: [http://www.iie.org](about:blank)

Karolinska Institute: [http://ki.se/en/](about:blank)

NRC: [www.nrc-cnrc.gc.ca/](about:blank)

PAHO: [http://www.paho.org/hq/](about:blank)

Rand Corporation: [www.rand.org](about:blank)

Science in Australia Gender equity: [http://www.sciencegenderequity.org.au/](about:blank)

Sexual and Gender Diversity Office (U of T): [https://sgdo.utoronto.ca/](about:blank)

Society for Canadian Women in Science and Technology: [http://www.scwist.ca](about:blank)

WHO: [http://www.who.int/en/](about:blank)

**General Grey**

Government of Canada: https://publications.gc.ca/site/eng/home.html

GreyNet International: [http://www.greylit.org](about:blank)

SIGLE (System for Information on Grey Literature in Europe): [http://www.opengrey.eu](about:blank)

National Technical Information Service (NTIS): [http://www.ntis.govhttp://www.ntis.gov](about:blank)

**Search engines**

Google Scholar: [https://scholar.google.com/intl/en/scholar/about.html](about:blank)

TRIP database: [http://www.tripdatabase.com/](about:blank)

**Thesis**

Center for Research Libraries Foreign Dissertation: <https://www.crl.edu/collections/topics/dissertations>

DART-Europe E-theses Portal: [http://www.dart-europe.eu/basic-search.phphttp://www.dart-europe.eu/basic-search.php](about:blank)

Electronic Theses Online Service (ETHOS) | British Library:

[http://ethos.bl.uk/Home.do;jsessionid=D96E9CF245B0FE0199DDDB94FF4BD2A7](about:blank)

Open access dissertations: [https://oatd.org](about:blank)

Thesis Canada Portal: http://www.bac-lac.gc.ca/eng/services/theses/Pages/theses-canada.aspx

**Conferences**

Gender Summit [https://gender-summit.com](about:blank)

The Canadian Coalition of Women in Engineering, Science, Trades and Technology

[http://www.ccwestt.org](about:blank)

**Other**

Association of Faculties of Medicine of Canada: [https://afmc.ca/](about:blank)

Association of American Medical Colleges: [https://www.aamc.org/](about:blank)

U15 | Group of Canadian Research Universities: [www.u15.ca](about:blank)

U of T Gender Report: http://www.faculty.utoronto.ca/reports/gender-equity-report/genderequity-pathways-to-leadership/

Institute for Gender and the Economy (GATE) at the Rotman School:

https://www.gendereconomy.org; [https://www.gendereconomy.org/research-briefs/](about:blank)

# Appendix 7 – L1 Screening Form for Titles and Abstracts

**Gender Equity 2: – Level 1 Cheat Sheet**

*Version 3*

**Review objective:** The goal is to conduct a living scoping review to identify existing interventions to optimise gender equity across a variety of disciplines to examine how these interventions can be implemented in academic health.

**Review questions:**

1. What interventions exist to optimize gender equity in all sectors and how can these interventions be implemented in academic health?

**Synthesi.SR link:** <https://synthesi.sr/web/login> **Project name: *Gender Equity 2*** ***– L1***

NOTES

- If you select ‘NO’ to any screening question, the study will be excluded, and the remaining questions can be skipped.
- If you select ‘UNCLEAR’ or ‘YES’ to all questions, the study will be included for Level 2 screening.
- If a study only has a title, select ‘UNCLEAR’ for all questions if the title mentions “gender” or “equity”.

| ***Question 1:** | **Does the study involve an intervention to promote gender equity among adults in any employment sector targeted to individuals, organizations, or systems?** | | |
| --- | --- | --- | --- |
| **RESPONSE** | **YES** | **NO** | **UNCLEAR** |
| Guidance notes | The intervention clearly targets at least one of the 3 pillars^1^ detailed below.  The intervention is targeted towards adults in any employment sector including academia, industry, law, government, education, business, and STEM | There is no discernible intervention for gender equity present | There is a lack of clarity about whether the intervention is to promote gender equity |
| ^1^Individuals – e.g. training in diversity, unconscious bias, writing grants, peer-reviewing grants, mentorship, coaching  Organizations – e.g. policies designed to address gender inequity, workplace code of conduct, implementation of a gender-blinding process of grant applications at the funding agency level, implementation of equity, diversity and inclusion committee at the department level, establishment of structures for mentorship at the institutional level, implementation of equity, diversity and inclusion action plan at the government level  Systems – e.g. legislation to publicly report salaries, legislation to mandate equitable representation on committees, pay equity | | | |

| **Question 2:** | **Is the study a relevant study design?** | | |
| --- | --- | --- | --- |
| **RESPONSE** | **YES** | **NO** | **UNCLEAR** |
| Guidance notes | Randomized controlled trials  Quasi randomized | All other study designs | Study design is not clearly reported  OR  If it is unclear whether adequate randomization took place |

| **Question 3:** | **Is this a potentially relevant study with the following formats?**  **[Flagging Question]** | | | | |
| --- | --- | --- | --- | --- | --- |
| **RESPONSE** | **Conference Abstract** | **Non-English Article** | **Protocol** | **Systematic Review** | **Unclear** |
| This is a flagging question for organizational purposes. It is only necessary to answer this question if the study is believed to fall into one of the above categories, otherwise please leave blank. | | | | | |

# Appendix 8 – L2 Screening Form for Full-Text Articles

**Gender Equity 2: – Level 2 Cheat Sheet**

*Version 4*

**Review objective:** The goal is to conduct a living scoping review to identify existing interventions to optimise gender equity across a variety of disciplines to examine how these interventions can be implemented in academic health.

**Review questions:**

1. What interventions exist to optimize gender equity in all sectors and how can these interventions be implemented in academic health?

**Synthesi.SR link:** <https://synthesi.sr/web/login> **Project name: *Gender Equity 2*** ***– L2***

NOTES

- If you select ‘NO’ to any screening question, the study will be excluded, and the remaining questions can be skipped.
- If you select ‘UNCLEAR’ or ‘YES’ to all questions, the study will be included for Level 2 screening.
- If a study only has a title, select ‘UNCLEAR’ for all questions if the title mentions “gender” or “equity”.

| ***Question 1:** | **The intervention is targeted towards an adult population in any employment or volunteer sector including academia, industry, law, government, education, business, and STEM.** | | |
| --- | --- | --- | --- |
| **RESPONSE** | **YES** | **NO** | **UNCLEAR** |
| Guidance notes | The intervention clearly targets at least one of the 3 pillars^1^ detailed below.  The intervention is targeted towards an adult population in any employment sector including academia, industry, law, government, education, business, volunteers, and STEM.  Interventions focusing on GE + LGBTQ2S+. | There is no discernible intervention for gender equity present.  The study population is students.  Interventions focusing on LGBTQ2S only. | There is a lack of clarity about whether the intervention is to promote gender equity. |
| ^1^Individuals – e.g. training in diversity, unconscious bias, writing grants, peer-reviewing grants, mentorship, coaching  Organizations – e.g. policies designed to address gender inequity, workplace code of conduct, implementation of a gender-blinding process of grant applications at the funding agency level, implementation of equity, diversity and inclusion committee at the department level, establishment of structures for mentorship at the institutional level, implementation of equity, diversity and inclusion action plan at the government level  Systems – e.g. legislation to publicly report salaries, legislation to mandate equitable representation on committees, pay equity | | | |

| **Question 2:** | **Is the study a relevant study design?** | | |
| --- | --- | --- | --- |
| **RESPONSE** | **YES** | **NO** | **UNCLEAR** |
| Guidance notes | Randomized controlled trials  Quasi randomized | All other study designs | Study design is not clearly reported  OR  If it is unclear whether adequate randomization took place |
| If a record is a Letter or Editorial but reports on a trial or data from a trial we would want to INCLUDE this type of record. | | | |

| **Question 3:** | **Does this study examine any gender equity outcomes?** | | |
| --- | --- | --- | --- |
| **RESPONSE** | **YES** | **NO** | **UNCLEAR** |
| Guidance notes | Any outcome related to the implementation of gender equity/inequity intervention, such as:  Changes in attitude  Changes in awareness  Changes in bias  Improvement in wage equality  Improvement of the gender balance in senior management  Etc. | There are no discernible outcomes related to gender equity present | Outcomes are not clearly reported  OR  If it is unclear whether the outcome is related to gender equity |

| **Question 4:** | **Is this a potentially relevant study with the following formats?**  **[Flagging Question]** | | | | |
| --- | --- | --- | --- | --- | --- |
| **RESPONSE** | **Conference Abstract** | **Non-English Article** | **Protocol** | **Systematic Review** | **Unclear** |
| This is a flagging question for organizational purposes. It is only necessary to answer this question if you answered YES or UNCLEAR to previous questions, and the study is believed to fall into one of the above categories, otherwise please leave blank. | | | | | |

# Appendix 9 – Data Abstraction Form

TAB 1. Study Characteristics

| **STUDY CHARACTERISTICS** | |
| --- | --- |
| **Excel column** | **Description** |
| RefID | Please fill in |
| Reviewer | Please fill in |
| Last name of first author | Enter the last name of the first author.  **Example:** Smith |
| Year of publication | Enter the year the study was first published.  **Example:** 2015 |
| Publishing source name | Enter the journal name or name of the publishing source (if the journal name is not available). |
| ublication type | Select the publication type.  Journal article  Report  Conference abstract  Results from trial registry  Dissertation/Thesis |
| Study design | Select the study design from the dropdown.  Randomized controlled trials (RCTs)  Cluster RCTs  Cross-over RCTs  Quasi-RCTs  Unclear  **Note:** Studies might not always explicitly state the design in which case select the best option based on the methods section. If unclear, please include any relevant information in the comment column at the end of the tab. |
| Country | Enter the country of conduct.  If the trial is a multi-site trial, ensure all the countries are listed (separated by commas). If the country of conduct is not clear, use the country from the first author’s affiliation. If the study doesn’t mention the country but mentions a region, enter the region (e.g., Sub-Saharan Africa).  **Example:** USA, Canada, Netherlands |
| Single or multicenter | From the dropdown, select whether the study is single center, multicenter, unclear or NR (not reported). |
| No. of centers in total | Enter the total number of centers partaking in the study. |
| Comment | Please enter any comments relative to study characteristics. |

TAB 2. Population

| **CHARACTERISTICS OF POPULATION** | |
| --- | --- |
| **Excel column** | **Description** |
| RefID | [Auto-populated based on previous entry] |
| Reviewer | [Auto-populated based on previous entry] |
| Participant inclusion criteria | Copy-paste the participant inclusion criteria for the study. |
| Participant exclusion criteria | Copy-paste the participant exclusion criteria for the study. |
| Setting | Copy and paste the description of the study setting.  **Example:** University classrooms, etc. |
| Overall sample size | Enter the total number of participants randomized (or quasi-randomized). |
| Intersectionality | Does the paper report on intersecting demographics? Please enter the combination of intersecting demographics in this column.  **Example:** gender + race (and not just gender and race separately), gender + race + disability |
| Race | Copy and paste the breakdown of participants’ race for the entire sample, if provided. |
| **Gender**  ***NOTE:*** *Please* *use the ‘gender’ section to report the genders of the population even if authors use the terms gender and sex interchangeably. The ‘sex’ column should be used to record if the authors specifically make the distinction between gender and sex in the study (e.g. authors collect data on the reported gender identity of the population AND they also record sex data from birth certificates).*  ***Note:*** *Please chart genders in the order they are reported in the study table*  *For the definition of gender and gender dimensions, please see “Additional information – Glossary, Gender” at the end of the document. A quick hint is that Gender is usually referred to as Woman / Man / Non-binary categories* | |
| Definition of Gender | How do the study authors define gender? Copy and paste from the text. |
| Gender group 1 | Enter the percent of gender group 1 for the entire sample, if available. |
| Gender group 2 | Enter the percent of gender group 2 for the entire sample, if available. |
| Gender groups 2-3 | Enter the percent of other specified gender or no specified gender. If there are multiple genders reported, separate using a semicolon. |
| Sex  If authors report population by gender, but also describe the sample using sex categories (e.g., from birth certificates), use this column to indicate this. A quick hint is that Sex is often referred to as Male / Female. | |
| Definition of sex | Please copy and paste the definition of sex as reported by the study authors, if available |
| Sex group 1 | Enter the percent of sex group 1 for the entire sample, if available. |
| Sex group 2 | Enter the percent of sex group 2 for the entire sample, if available. |
| Age value | Enter the overall age of participants in the sample. If age is reported as a range category (e.g., 35-50 years), enter the relevant category and accompanying percentage with a colon in between (e.g., 35-50: 50%). If there are multiple range categories, separate using a semicolon (e.g., 35-50: 50%; 50-65: 50%)) |
| Age measure | Select how age is reported for the overall sample, if a mean and a median is provided, please abstract both where possible:  Mean  Median  A single range  Range Categories  Unclear  NR |
| Age variance value | Enter the value of the variance reported.  **Example:** SD: 14.3 (if mean or median were the measures); Range: 35-50 (if a single range for the sample was reported) |
| ge variance type | Select from the dropdown menu:  Standard deviation (SD)  Standard error (SE)  Range  Interquartile range (IQR)  95% CI  Unclear  NR |
| Tx1_Name | Enter the name of the intervention allocated to the treatment arm.  **Example:** Workshop sessions |
| Tx1_Place of residence | Copy and paste the breakdown of places of residence (e.g., rural, urban, suburban, long-term care, congregate setting etc). |
| Race  For the definition of race, please see “Additional information – Glossary, Race” at the end of the document | |
| Tx1_Race | Copy and paste the breakdown of races. |
| Tx1_Occupation | Copy and paste the breakdown of occupations. |
| Tx1_% Gender group 1 | Enter the percent of gender group 1. |
| Tx1_% Gender group 2 | Enter the percent of gender group 2. |
| Tx1_% Gender group 2-3 | Enter the percent of other specified gender or no specified gender. If there are multiple genders reported, separate using a semicolon. |
| Tx1_% Sex group 1 | Enter the percent of sex group 1. |
| Tx1_% Sex group 2 | Enter the percent of sex group 2. |
| Religion  For the definition of religion, please see “Additional information – Glossary, Religious background” at the end of the document | |
| Tx1_ Religious backgrounds | Copy and paste the breakdown of religious backgrounds. |
| Tx1_Education | Copy and paste the breakdown of educational backgrounds. |
| Socioeconomic status  For the definition of socioeconomic status, please see “Additional information – Glossary, Socioeconomic status” at the end of the document | |
| Tx1_ Socioeconomic status | Copy and paste the breakdown of participants’ socioeconomic status. |
| Social capital  For the definition of social capital, please see “Additional information – Glossary, Social capital” at the end of the document | |
| Tx1_Social capital | Copy and paste the breakdown of participants’ social capital categories. |
| Disability  For the definition of disability, please see “Additional information – Glossary, Disability” at the end of the document | |
| Tx1_Disability | Copy and paste the breakdown of participants’ disability categories. |
| Comment | Enter any relevant comments for the participant characteristics that are not captured elsewhere. |
| *Please add more columns if there are additional arms.* | |

TAB 3. Intervention Characteristics

| **INTERVENTION CHARACTERISTICS** | |
| --- | --- |
| **Excel column** | **Description** |
| RefID | [Auto-populated based on previous entry] |
| Reviewer | [Auto-populated based on previous entry] |
| x1_name | Enter the name of the intervention allocated to the treatment arm.  **Example:** Workshop sessions |
| Tx1_Description | Enter a description of what the intervention entailed (copy and paste description from paper) |
| Tx1_intersectionality | Does the intervention consider intersectionality? Enter a description of how the intervention considers intersectionality of participants. If the intervention does not appear to consider intersectionality, please put NR. |
| Intersectionality  For the definition of intersectionality, please see “Additional information – Glossary, Intersectionality” at the end of the document | |
| x1_sample size | Enter the number of participants randomized to this study arm that received the intervention. |
| Tx1_duration | Enter the duration of the intervention if applicable. If the intervention was a single day event, enter ‘NA’. |
| Tx1_lost to follow up | Enter the number of participants lost to follow up in this study arm as indicated by the author. |
| Tx1_age value | Enter the overall age of participants in this intervention arm. If age is reported as a category (e.g., 35-50 years), enter the relevant category and accompanying value with a colon in between (e.g., 35-50: 50%). If there are multiple categories, separate using a semicolon (e.g., 35-50: 50%; 50-65: 50%)) |
| Tx1_age measure | Select how age is reported:  Mean  Median  Range  Bracket  Unclear  NR |
| Tx1_ age variance value | Enter the value of the variance reported.  **Example:** SD: 14.3; Range: 35-50 |
| x1_ age variance type | Select from the dropdown menu:  Standard deviation (SD)  Standard error (SE)  Range  Interquartile range (IQR)  95% CI  NR |
| Comments | Enter any comments relevant to the intervention. |
| *Please add more columns if there are additional arms.* | |

TAB 4. Statistical Model Data

| **Excel column** | **Description** |
| --- | --- |
| RefID | Enter the refID of the study being abstracted |
| Reviewer | Enter your initials |
| Tx1_name | Enter the name of the intervention allocated to the Tx1 arm.  **Example**: Workshop sessions |
| Tx2_name | Enter the name of the intervention allocated to the Tx2 arm.  **Example**: No intervention |
| *If there are multiple interventions or combinations of interventions taking place in the study, please capture this in a second, third, etc. row.* | |
| Outcome measure | Enter the relevant outcomes  **Example:** Culture change (CCWAS score) |
| Outcome measurement time point | Select the time point for which you are abstracting results from the dropdown menu  **Options include:** Baseline, Follow-up  *Note: For each outcome measure, the baseline values and follow-up values will be captured in separate rows* |
| Measure of effect/association type | Enter the name of the measure of effect/association (e.g., adjusted odds ratio, adjusted rate ratio). Only enter unadjusted effect size if adjusted is not reported.  *Note: If there are more than 2 treatment arms, there may be 1v2, 1v3, etc. To capture this, please add additional rows for the same study to reflect the multiple outcomes, values, participants, etc.* |
| Measure of effect/association value | Enter the value for the measure of effect/association. |
| 95% CI | Enter the 95% CI for the adjusted OR. |
| Analysis method | Enter in the analysis methods used.  **Example:** Multiple logistic/log binomial/Cox proportional hazards regression |
| Confounders / variables controlled for in model | List any confounders or variables which were controlled for in the model. Copy and paste any description of confounders/controls from the paper. |
| Number of participants | Enter the total number of participants included in the model. |
| Qualitative Evidence | Report any results collected narratively. |
| Comments | Enter any comments relevant to the treatment arm comparisons. For example, if matching was used instead of adjustment in a quasi-RCT. |

ADDITIONAL INFORMATION USED IN SCREENING: Glossary

**Disability:** The United Nations’ Convention on the Rights of Persons with Disabilities (CRPD) recognizes “disability is an evolving concept and that disability results from the interaction between persons with impairments and attitudinal and environmental barriers that hinder their full and effective participation in society on an equal basis with others.” … Defining disability is a complex, evolving matter. The term “disability” covers a broad range and degree of conditions. A disability may have been present at birth, caused by an accident, or developed over time. Section 10 of the Code defines “disability” as^3^:

- any degree of physical disability, infirmity, malformation or disfigurement that is caused by bodily injury, birth defect or illness and, without limiting the generality of the foregoing, includes diabetes mellitus, epilepsy, a brain injury, any degree of paralysis, amputation, lack of physical co-ordination, blindness or visual impediment, deafness or hearing impediment, muteness or speech impediment, or physical reliance on a guide dog or other animal or on a wheelchair or other remedial appliance or device,
- a condition of mental impairment or a developmental disability,
- a learning disability, or a dysfunction in one or more of the processes involved in understanding or using symbols or spoken language,
- a mental disorder, or
- an injury or disability for which benefits were claimed or received under the insurance plan established under the Workplace Safety and Insurance Act, 1997.^3^

**Equity:** Equity means fairness; people of all identities being treated fairly. It means ensuring that the processes for allocating resources and decision-making are fair to all and do not discriminate on the basis of identity. There is a need to put measures in place to eliminate discrimination and inequalities which have been well described and reported and ensure, to the best degree possible, equal opportunities. Equity is needed to achieve equality. For example, treating people as equals in an environment in which historical and systemic disadvantages prevent people from operating as equals can be inequitable – it lacks the fairness of a truly equitable situation.”^1^

**Ethnicity:** Ethnicity denotes groups, such as Irish, Fijian, or Sioux, etc. that share a common identity-based ancestry, language, or culture. It is often based on religion, beliefs, and customs as well as memories of migration or colonization (Cornell & Hartmann, 2007). In scientific analysis, it can be important to distinguish, however loosely, between race and ethnicity. Biological anthropologist Fatimah Jackson (2003) provides a pertinent example of cultural practices being misread as biological differences. Microethnic groups living in the Mississippi Delta, she writes, use sassafras in traditional cooking. Sassafras increases susceptibility to pancreatic cancer. Medical practitioners who do not carefully disaggregate cultural and biological traits might interpret a geographic cluster of pancreatic cancer as related to a genetic or racial trait when, in fact, the disease is produced by cultural practices—in this case, shared culinary habits. ^5^

**Gender:** Gender isn’t about someone’s anatomy, it is about who they know them self to be. There are many different gender identities, including self-identified as being male, female, transgender, gender neutral, non-binary, agender, pangender, genderqueer, two-spirit, third gender, and all, none or a combination of these. ^8^

- **Cisgender:** Cisgender is a term to describe someone whose gender matches what they were assigned at birth. For example, they were assigned female at birth based on being born with a vagina and know themselves to be female. ^8^
- **Gender binary**: The social classification system that recognizes only two genders; this system requires everyone to be raised as a man or a woman, which is dependent on the gender assigned at birth; “The classification of gender as two opposite genders: male and female”^7^
- **Gender fluid:** Gender fluid may refer to a gender which varies over time. Someone who identifies as gender fluid may fluctuate between genders or express multiple genders at the same time. Their gender may also vary at random or vary in response to different circumstances.^8^
- **Gender-nonconforming people:** Individuals who do not adhere to gender binaries. ^8^
- **Gender neutral:** Someone who feels they are neither male or female may identify as gender neutral. ^8^
- **Genderqueer people:** Someone may identify as genderqueer if their gender identity is neither male nor female, is between or beyond genders, or is some combination of genders. This identity is often related to or in reaction to the social construction of gender, gender stereotypes and the gender binary system. ^8^
- **Lesbian:** a woman who is sexually, romantically, and/or emotionally attracted to women. ^8^
- **Non-binary:** Someone who does not identify as a man or a woman, or solely as one of those two genders. It’s often used as an umbrella term for identities that fall outside the male/female gender binary. Being non-binary means different things to different people, so this definition is purposely broad.^8^
- **Transgender:** Someone whose gender identity is different from the one they were assigned at birth might identify as transgender. Sometimes trans or transgender gets used as an umbrella term for gender diverse people. However, not everyone uses it to describe themselves. When in doubt ask, and always honour someone’s personal terms when it comes to gender identities. What do we mean assigned at birth? Think about one of the first things the doctor (or midwife) says when someone is born, “It’s a girl” or “It’s a boy.” This is gender assignment and it is based on an assumption that someone’s genitals match their gender. However, we know this isn’t always the case and that each of us gets to decide what gender we know our selves to be. It may not align with the genitals we are born with or be part of the gender binary (male-female). For example, someone may be born with a vagina but know themselves to be male. ^8^
- **Trans man:** A man whose gender assigned at birth differs from their current gender as a man. Not all of us are FTM (female-to-male) or were assigned female at birth. Many trans men transition to become more comfortable and at one with their authentic selves.^7^
- **Two Spirit:** “The contemporary term Two Spirit was first coined in 1990 at the 3rd annual Native American and Canadian Aboriginal LGBT people gathering in Winnipeg. In creating the term, the founding group wanted to reflect the historical acceptance of gender-variant peoples and diverse sexual identities within Indigenous communities in pre-contact times. ^6^
  - Two Spirit is meant to be an umbrella term that points to the important roles that Two Spirit people held prior to colonization; however, as an umbrella term, specific teachings, roles, meanings, and language must come from the community. For example a Cree ‘Two Spirit’ person from the plains area could go by aayahkwew (roughly translates to “neither man nor woman”) while a Mohawk ‘Two Spirit’ person could go by Onón:wat (I have the pattern of two spirits inside my body). Furthermore, the teachings, roles, and responsibilities for a Two Spirit person differs from community to community.
  - The identity itself was introduced by the Elder Myra Laramee through a vision she had prior to the 1990 gathering in Winnipeg. Within this vision, Myra shared the vision she had of her Anishinaabemowin name of niizh manidoowag; which roughly translates to having the ability to be neutral through the lens of having both a feminine spirit and masculine spirit within one's body.
  - Being Two Spirit is a very fluid identity and each tribe and Indigenous person has their own understanding of what it means to live and be Two Spirit. One important element to note however is that the identity is specific to being Indigenous, in that the identity is a direct acknowledgement of the disruption of Two Spirit teachings that took place when first-contact between Indigenous peoples and settlers was made and the ongoing impact of colonization. ^6^
- **Trans women:** A woman who was mistakenly assigned male at birth and, as a consequence of that mistake, was likely raised as boy.^7^
- **Queer**: An umbrella term to refer to all LGBTQ+ people. It is also a non-binary term used by individuals who see their sexual orientation and/or gender identity as fluid. ^6^

**Intersectionality:** The interconnected nature of social categorizations such as race, class, and gender as they apply to a given individual or group, regarded as creating overlapping and interdependent systems of discrimination or disadvantage.

**Race:** Race is a powerful social category forged historically through oppression, slavery, and conquest. Most geneticists agree that racial taxonomies at the DNA level are invalid. Genetic differences within any designated racial group are often greater than differences between racial groups. Most genetic markers do not differ sufficiently by race to be useful in medical research.^5^

- **Race as a Social Category:** Humans vary remarkably in wealth, exposure to environmental toxins, and access to medicine. These factors can create health disparities. Krieger (2000) describes disparities that result from racial discrimination as “biological expressions of race relations.” African Americans, for example, have higher rates of mortality than other racial groups for 8 of the top 10 causes of death in the U.S. (Race, Ethnicity, and Genetics Working Group, 2005). Although these disparities can be explained in part by social class, they are not reducible to class distinctions.^5^

**Religious background:** Religious beliefs are a generalized system of ideas and values that shape how members of a religious group come to understand the world around them. Religion contributes to inequities when access to certain goods/services/benefits is limited for a subgroup of the population because of their religious affiliation or lack of religion).^4^

**Socioeconomic Status:** Socioeconomic status is the position of an individual or group on the socioeconomic scale, which is determined by a combination of social and economic factors such as income, amount and kind of education, type and prestige of occupation.

**Social capital:** Social capital refers to social relationships and networks. It includes interpersonal trust between members of a community, civic participation, and the willingness of members of a community to assist each other and facilitate the realization of collective community goals and the strength of their political connections, which can facilitate access to services.^2^

**References:**

1. Natural Sciences and Engineering Research Council of Canada. "Guide for applicants: Considering equity, diversity, and inclusion in your application." (2017). Available at: https://www.nserc-crsng.gc.ca/_doc/EDI/Guide_for_Applicants_EN.pdf
2. O'Neill J, Tabish H, Welch V, Petticrew M, Pottie K, Clarke M, Evans T, Pardo Pardo J, Waters E, White H, Tugwell P. Applying an equity lens to interventions: using PROGRESS ensures consideration of socially stratifying factors to illuminate inequities in health. J Clin Epidemiol. 2014 Jan;67(1):56-64. doi: 10.1016/j.jclinepi.2013.08.005. Epub 2013 Nov 1. PMID: 24189091.
3. Ontario Human Rights Commission. What is disability? Available at: http://www.ohrc.on.ca/en/policy-ableism-and-discrimination-based-disability/2-what-disability
4. Little W, McGivern R, Kerins N. Introduction to sociology-2nd Canadian edition. BC Campus; 2016. Available at: https://opentextbc.ca/introductiontosociology2ndedition/chapter/chapter-15-religion/
5. Race & Ethnicity. Gendered Innovations in Science, health & Medicine, Engineering, and Environment, Stanford University. Available at: https://genderedinnovations.stanford.edu/terms/race.html
6. Two Spirit. Resource of University of Saskatoon. Available at: https://www.outsaskatoon.ca/two_spirit1
7. Queer undefined a crowdsourced lgbtq+ dictionary. Available at: https://www.queerundefined.com/search/trans%20guy
8. Teen Talk. SERC: Sexuality Education Resource Centre, Manitoba. Available at: https://teentalk.ca/learn-about/gender-identity/
9. WAAC online resource. Available at: https://waaids.com/item/736-sexuality.html

#

# Appendix 10 – Closely Related but Ultimately Excluded Studies

| **Reference** | **Reason for exclusion** |
| --- | --- |
| Krishnan, S., Gambhir, S., Luecke, E., & Jagannathan, L. (2016). Impact of a workplace intervention on attitudes and practices related to gender equity in Bengaluru, India. *Global Public Health*, *11*(9), 1169-1184. | Not a RCT or quasi-randomized trial. |
| Kalra, P., & Boukes, M. (2021). Curbing journalistic gender bias: How activating awareness of gender bias in Indian journalists affects their reporting. *Journalism Practice*, *15*(5), 651-668. | Majority (74%) of the sample is students. |
| Karim, S., Gilligan, M. J., Blair, R., & Beardsley, K. (2018). International gender balancing reforms in postconflict countries: Lab-in-the-field evidence from the liberian national police. *International Studies Quarterly*, *62*(3), 618-631. | Not a RCT or quasi-randomized trial |
| Zinovyeva, N., & Bagues, M. (2010). Does gender matter for academic promotion? Evidence from a randomized natural experiment. | Not a RCT or quasi-randomized trial |
| Brescoll, V. L., & Uhlmann, E. L. (2008). Can an angry woman get ahead? Status conferral, gender, and expression of emotion in the workplace. *Psychological science*, *19*(3), 268-275. | Not a RCT or quasi-randomized trial |
| Glick, P., Zion, C., & Nelson, C. (1988). What mediates sex discrimination in hiring decisions?. *Journal of Personality and Social Psychology*, *55*(2), 178. | Not a RCT or quasi-randomized trial |
| Rosen, B., & Mericle, M. F. (1979). Influence of strong versus weak fair employment policies and applicant's sex on selection decisions and salary recommendations in a management simulation. *Journal of Applied Psychology*, *64*(4), 435. | Not a RCT or quasi-randomized trial |

# Appendix 11 – Study Characteristics

| **Author, year of publication** | **Article Title** | **Journal Name** | **Country** | **Study design** | **Setting** | **Multicenter vs. Single Site** | **Number of Participants** |
| --- | --- | --- | --- | --- | --- | --- | --- |
| **Included trials** | | | | | | | |
| Bapna, 2021 | Interventions for Improving Professional Networking for Women: Experimental Evidence from the IT Sector | Management Information Systems Quarterly | USA | RCT | Corporate | Single | 958 |
| Bates, 2019 | The Gender By Us® Toolkit:  A Pilot Study of an Intervention  to Disrupt Implicit Gender Bias | Journal of Women and Social Work | USA | RCT | Workplace | Single | 23 |
| Brady, 2015 (study 1/2) | It's fair for us: Diversity structures cause women to  legitimize discrimination | Journal of Experimental Social Psychology | USA | RCT | Corporate | Single | 119 |
| Brady, 2015 (study 2/2) | It's fair for us: Diversity structures cause women to  legitimize discrimination | Journal of Experimental Social Psychology | USA | RCT | Academic | Single | 178 |
| Bulte, 2017 | Do Gender and Business Trainings Affect Business  Outcomes? Experimental Evidence from Vietnam | Management Science | Vietnam | RCT | Urban | Multicenter | 4041 |
| Chinen, 2017 | Can Teacher Training Programs Influence Gender Norms? Mixed-Methods Experimental  Evidence from Northern Uganda | Journal on Education in Emergencies | Uganda | Cluster RCT | Urban & Rural | Multicenter | 105 schools (916 teachers) |
| Cook, 2019 | Gender quotas increase the equality and  effectiveness of climate policy interventions | Nature Climate Change | Indonesia  Peru  Tanzania | Unclear | Rural | Multicenter | 440 |
| Dahl, 2018 | Does Integration Change Gender Attitudes? The Effect of Randomly Assigning Women to Traditionally Male Teams | National Bureau of Economic Research | Norway | RCT | Military | Multicenter | 900 |
| Ginter, 2020 | Can Mentoring Help Female Assistant Professors in Economics? An Evaluation by Randomized Trial | NBER working paper series | USA | RCT | Academic | NR | 365 |
| Grisso, 2015 | A Randomized Controlled Trial to Improve  the Success of Women Assistant Professors | Journal of Women's Health | USA | Cluster RCT | Academic | Multicenter | 25 departments/divsions (clusters), 134 participants (women assistant professors) |
| Huis, 2019* | The impact of husbands' involvement in goalsetting  training on women's empowerment: First  evidence from an intervention among female  microfinance borrowers in Sri Lanka | J Community Appl Soc Psychol. | Sri Lanka | RCT | Workplace – Microfinance | Single | 74 couples |
| Huis, 2019 | Impacts of the Gender and Entrepreneurship Together Ahead  (GET Ahead) training on empowerment of female microfinance borrowers in Northern Vietnam | World Development | Vietnam | Cluster RCT | Workplace – Microfinance | Multicenter | 4041 + 315 additional |
| Ismayilova, 2017 | An Integrated Approach to Increasing Women’s Empowerment Status and  Reducing Domestic Violence: Results of a Cluster-Randomized Controlled  Trial in a West African Country | Psychology of Violence | Burkina Faso | Cluster RCT | Workplace – Microfinance | Multicenter | 12 villages, 360 women |
| Matsutaka, 2022 | Psychology of Sexual Orientation and Gender  Diversity  Development and Evaluation of a Training Program to Reduce Homophobia  and Transphobia Among Human Resource Staff and Health Professionals in  the Workplace: A Randomized Controlled Trial | Psychology of Sexual Orientation and Gender Diversity | Japan | RCT | Corporate | Single | 39 |
| O’Meara, 2018 | Undoing disparities in faculty workloads: A  randomized trial experiment | PLoS ONE | USA | RCT | Academic | Multicenter | 472 |
| Paek, 2022 | Manager gender and changing attitudes toward  schedule control: evidence from the Work, Family,  and Health Study | Community, Work & Family | USA | RCT | Corporate | Unclear | 209 |
| Peterson, 2019 | Mitigating gender bias in student evaluations  of teaching | PLOS One | USA | RCT | Academic | Single | 247 |
| Rivera, 2019 | Scaling Down Inequality:  Rating Scales, Gender Bias, and  the Architecture of Evaluation | American Sociological Review | USA | Quasi-RCT | Academic | Single | 369 |
| Shankar, 2015 | Agency-Based Empowerment Training Enhances  Sales Capacity of Female Energy Entrepreneurs in  Kenya | Journal of Health Communications | Kenya | RCT | Urban & Rural | Multicenter | 300 |
| Smith, 2015 | Now Hiring! Empirically Testing a Three-Step Intervention to Increase Faculty Gender Diversity in STEM | BioScience | USA | RCT | Academic | Single | NR |
| Warren, 2017 | A Video Intervention for Professionals Working with Transgender and Gender  Nonconforming Older Adults | University of Missouri-St. Louis; ProQuest | USA | RCT | Workplace | Multicenter | 155 |
| Webb, 2012 | Gaining control over responses to implicit attitude  tests: Implementation intentions engender fast  responses on attitude-incongruent trials | British Journal of Social Psychology | Republic of Ireland | RCT | Corporate | Multicenter | 40 |
| Wiseman, 1979 | Attitude and behavioral change in academic advisors at Montana State University : sex role stereotyping and sexual bias in vocational choice | Montana State University | USA | RCT | Academic | Single | 49 |
| Woolnough, 2007 | A Longitudinal Study to Investigate the Impact of a Career Development and Mentoring Programme on Female Mental Health Nurses | ProQuest | UK | RCT | Healthcare | Multicenter | 54 |
| ***Companion reports** | | | | | | | |
| Huis, 2019 (companion report) | The impact of husbands' involvement in goalsetting  training on women's empowerment: First  evidence from an intervention among female  microfinance borrowers in Sri Lanka | J Community Appl Soc Psychol. | Sri Lanka | RCT | Workplace – Microfinance | Single | 68 couples |

# Appendix 12 – Participant Characteristics

| **Author, year** | **Intersectionality** | **Race** | **Definition of Gender** | **Age Value (years)** | **Age Measure** | **Age Variance Value** | **Age Variance Type** |
| --- | --- | --- | --- | --- | --- | --- | --- |
| **Included trials** | | | | | | | |
| Bapna, 2021 | NR | NR | NR | NR | NR | NR | NR |
| Bates, 2019 | NR | Overall:  Asian (13%); African American (13%); White, Caucasian (65%); Other (9%) | Male/Female | NR | NR | NR | NR |
| Brady, 2015  (study 1/2) | NR | White American (85.8%); African American (4.4%); Asian American (3.5%); Latino/Hispanic American (1.8%); Native American (.9%); Multiracial or Other (3.5%) | Women | 32.28 | Mean | 12.27 | SD |
| Brady, 2015  (study 2/2) | NR | White American (25.5%); African American (2.1%); Asian American (53.2%); Hispanic American (7.1%); Native American (0.9%); Multiracial or Other (12.1%) | Women | 18.76 | Mean | 1.13 | SD |
| Bulte, 2017 | NR | NR | Male/Female | 43.77 | Mean | 10.33 | SD |
| Chinen, 2017 | NR | NR | Group 1 "female"; Group 2 "male" | NR | NR | NR | NR |
| Cook, 2019 | NR | NR | NR | 38.87 | Mean | 11.86 | SD |
| Dahl, 2018 | NR | NR | NR | NR, but inclusion is 18 to 19 years | Range | NR | NR |
| Ginter, 2020 | NR | NR | All participants "women" | NR | NR | NR | NR |
| Grisso, 2015 | NR | Overall:  African American (7.6%); White (60.3%); Asian (27.5%); Hispanic/other (4.6%) | Women | 40 | Median | 37-44 | Q1-Q3 |
| Huis, 2019* | NR | NR | NR | 36.27 | Mean | 7.66 | SD |
| Huis, 2019 | NR | NR | Female | NR | Mean | NR | SD |
| Ismayilova, 2017 | NR | Ethnicity: Mossi (98.61%); Peuhl (1.11%); Other - Gourmantche (0.28%) | Women | 37.21 | Mean | 35.68 to 38.74 | 95% CI |
| Matsutaka, 2022 | Gender identity; Sexual orientation | NR | Male/Female | 51.06 intervention, 46.76 control | Mean | 5.57 intervention, 8.41 control | SD |
| O’Meara, 2018 | NR | Overall:  American Indian or Alaska Native (0.7%); Asian (9.6%); Black/African American (10.7%); White (75.1%); Multi-Racial (3.9%) | NR | NR | NR | NR | NR |
| Paek, 2022 | NR | Overall:  White (80%); Asian (17%); Black and Hispanic (3%) | Women/Men | NR | NR | NR | NR |
| Peterson, 2019 | NR | NR | NR | NR | NR | NR | NR |
| Rivera, 2019 | NR | NR | NR | NR | NR | NR | NR |
| Shankar, 2015 | NR | NR | Male/Female | 14% less than 25, 38% 25-34, 25% 35-44, 23% 45+ | NA | NA | NA |
| Smith, 2015 | NR | NR | NR | NR | NR | NR | NR |
| Warren, 2017 | NR | Overall:  White/Caucasian (82.6%); Biracial/Multiracial (7.1%); Hispanic/Latino(5.2%); Black/African American (4.5%); Asian/Pacific Islander (0.6%) | NR | 45.19 | Mean | 13.89 | SD |
| Webb, 2012 | NR | NR | NR | 43.45 | Mean | 8.28 | SD |
| Wiseman, 1979 | NR | NR | Group 1 "female"; Group 2 "male" | NR | NR | NR | NR |
| Woolnough, 2007 | NR | Overall:  British – White (90.7%); British – Afro Caribbean (1.9%); Mixed race (1.9%); Indian (3.7%) | NR | NR | NR | NR | NR |
| ***Companion reports** | | | | | | | |
| Huis, 2019 | NR | NR | NR | 37.27 | Mean | 6.95 | SD |
| NA: Not Applicable  NR: Not Reported | | | | | | | |

# Appendix 13 – PROGRESS^1^ Plus Table

| **Author, year** | **Place of Residence** | **Race** | **Occupation** | **Gender/ Sex** | **Religion** | **Education** | **Socioeconomic Status** | | **Social Capital** | **Plus** |
| --- | --- | --- | --- | --- | --- | --- | --- | --- | --- | --- |
| **Included trials** | | | | | | | | | | |
| Bapna, 2021 | NR | NR | Tx1, Tx2, Tx3: IT sector (100%) | Tx1, Tx2, and Tx3: Group 1 – Women (27.6%), Group 2 – Men (72.4%) | NR | Tx4: Individuals who registered to attend the conference had 717 distinct job titles from 456 distinct companies in IT sector | | NR | NR | NR |
| Bates, 2019 | NR | NR | Tx1 and Tx2: Full-time employment | Tx1: Group 1 – Male (27%), Group 2 – Female (73%)  Tx2: Group 1 – Male (17%), Group 2 – Female (83%) | NR | Tx1: Some college, no degree 1 (9%), Associates degree 1 (9%), Bachelor’s degree 5 (46%), Graduate degree 2 (18%), Professional degree 2 (18%)  Tx2: Some college, no degree 2 (17%), Associates degree 1 (8%), Bachelor’s degree 1 (8%), Graduate degree 7 (53%), Professional degree 1 (8%) | | Tx1: Income – Less than US$25,000 3 (27%), US$25,000–US$34,999 1 (9%),  US$35,000–US$49,999 1 (9%), US$50,000–US$79,999 4 (36%),  US$80,000–US$99,999 1 (9%),  US$100,000–US$149,999 1 (9%)  Tx2: Income – Less than US$25,000 1 (8%), US$25,000–US$34,999 0 (0%), US$35,000–US$49,999 4 (33%), US$50,000–US$79,999 7 (58%), US$80,000–US$99,999 0 (0%), US$100,000–US$149,999 0 (0%) | NR | NR |
| Brady, 2015  (1/2) | NR | NR | NR | Tx1, Tx2, and Tx3: Group 1 – 100% | NR | NR | | NR | NR | Tx3: Only women in this study |
| Brady, 2015  (2/2) | NR | NR | NR | Tx1, Tx2, and Tx3: Group 1 – 100% | NR | NR | | NR | NR | Tx3: Only women in this study |
| Bulte, 2017 | Tx1, Tx2 and Tx3: Hanoi | Tx1, Tx2 and Tx3: Kihn (ethnic group) | Tx1, Tx2 and Tx3: 33% of our respondents are involved in nonfarm economic activities, and 78% of our respondents are involved in agricultural activities (production and sales) | Tx1, Tx2 and Tx3: Group 1 – Female (100%) | NR | Tx1, Tx2 and Tx3: Schooling years = 6.82 for all groups | | Tx1, Tx2 and Tx3: Poor | NR | NR |
| Chinen, 2017 | Tx1, Tx2, and Tx3: Karamoja | Tx1: Ethnicity – Ethur (43%), Bokora Karimojong (9%), Other (26%)  Tx2: Ethnicity – Ethur (37%), Bokora Karimojong (13%), Other (29%)  Tx3: Ethnicity – Ethur (35%), Bokora Karimojong (11%), Other (32%) | Tx1, Tx2, and Tx3: Teachers | Tx1: Group 1 – Female (24%), Group 2 – Male (76%)  Tx2: Group 1 – Female (27%), Group 2 – (73%)  Tx3: Group 1 – Female (25%), Group 2 – Male (75%) | Tx1: Catholic (67%), Protestant (23%), Other (10%)  Tx2: Catholic (62%), Protestant (27%), Other (12%)  Tx3: Catholic (61%), Protestant (28%), Other (11%) | NR | | NR | NR | NR |
| Cook, 2019 | NR | NR | NR | Tx1: Group 1 – Female (76%), Group 2 – Male (24%)  Tx2: Group 1 – Female (33%), Group 2 – Male (67%) | NR | Tx1: Years of formal education (mean, SD) – 7.2 SD 3.06 | | NR | NR | NR |
| Dahl, 2018 | Tx1 and Tx2: Military boot camp | NR | Tx1 and Tx2: Military conscript | Tx2: Group 1 – Male (100%), Group 2 – Female (0%) | NR | NR | | NR | NR | NR |
| Ginter, 2020 | NR | NR | Tx1 and Tx2: Junior Faculty | Tx1 and Tx2: Group 1 – 100%, Group 2 – 0% | NR | NR | | NR | NR | NR |
| Grisso, 2015 | NR | NR | Tx1 and Tx2: Assistant Professor | Tx1 and Tx2: Group 1 - Women (100%) | NR | NR | | NR | NR | NR |
| Huis, 2019* | NR | NR | Tx1, Tx2, Tx3: Female entrepreneur plus husband - 76.4% of the couples worked together as a couple in their business (e.g., agriculture, selling products) | Tx1, Tx2, and Tx3: Group 1 – Female (100%), Group 2 – Male (NA) | NR | NR | | NR | NR | NR |
| Huis, 2019 | NR | NR | Tx1, Tx2, and Tx3: Entrepreneur | Tx1, Tx2, and Tx3: Group 1 – 100% | NR | Tx1: Mean educational Level of 1.93  Tx2: Mean educational Level of 1.97  Tx3: Mean educational Level of 1.95 | | Tx1, Tx2, and Tx3: Poor (implied) | NR | Tx3: Only women in this study |
| Ismayilova, 2017 | NR | Tx1: Ethnicity – Mossi (99.17%), Peuhl (0.83%), Other (Gourmantche) (0%)  Tx2: : Ethnicity – Mossi (97.5%), Peuhl (2.5%), Other (Gourmantche) (0%)  Tx3: Ethnicity – Mossi (99.17%), Peuhl (0%), Other (Gourmantche) (0.83%) | Tx1, Tx2, and Tx3: : Nearly 100% of the women used a portion of their seed capital grant to invest in livestock and most also invested in some type of petty commerce | Tx1, Tx2, Tx3: Group 1 – 100% | Tx1: Christian 3.33%, Muslim 96.67%  Tx2: Christian 1.67%, Muslim 98.33%  Tx3: Christian 0.83%, Muslim 99.17% | Tx1: Mother's years of education, mean = 0.19  Tx2: Mother's years of education, mean = 0.26  Tx3: Mother's years of education, mean = 0.28 | | Tx1, Tx2, and Tx3: Ultrapoor, Ultrapoverty (lowest level of poverty or poorest of the poor) was determined based on a participatory wealth ranking (PWR) exercise developed and used by the implementing partner, Trickle Up (TU) organization | NR | Tx3: Only women in this study |
| Matsutaka, 2022 | NR | NR | Tx1: Personnel and labor relations staff 8 (44.4%), Diversity promotion staff 2 (11.1%), Occupational health staff 5 (27.8%), Manager 3 (16.7%)  Tx2: Personnel and labor relations staff 7 (33.3%), Diversity promotion staff 4 (19.0%), Occupational health staff 5 (23.8%), Manager 5 (23.8%) | Tx1: Group 1 – Female (55.6%), Group 2 - Male (44.4%)  Tx2: Group 1- Female (90.5%), Group 2 – Male (9.5%) | NR | NR | | Tx1: Employment status – Full-time work 17 (94.4%), Part-time work 1 (5.6%)  Tx2: Employment status – Full-time work 21 (100%); Part-time work 0 (0.0%) | NR | NR |
| O’Meara, 2018 | NR | NR | NR | NR | NR | Tx3: Total sample: Rank - Assistant Professors (21.2%), Associate Professors (29.8%), Full Professors (28.9%), Non Tenure-Track Faculty (20.1%)  Tx4: Total sample: Rank - Assistant Professors 21.2% Associate Professors 29.8%, Full Professors 28.9%, Non Tenure-Track Faculty 20.1% | | NR | NR | NR |
| Paek, 2022 | NR | NR | Tx1 and Tx2: Managers at a Fortune 500 IT company | Tx1: Group 1 – Female (40.6%), Group 2 – Male (59.4%)  Tx2: Group 1 – Female (26.2%), Group 2 – Male (73.8%) | NR | NR | | NR | NR | NR |
| Peterson, 2019 | NR | NR | NR | Tx2: Group 1 – 64.2% | NR | NR | | NR | NR | NR |
| Rivera, 2019 | NR | NR | NR | NR | NR | NR | | NR | NR | NR |
| Shankar, 2015 | Tx1 and Tx2: Urban slum in Kayole, Nairobi, Rural Tigania East in Meru County in central Kenya | NR | Tx1 and Tx2: Entrepreneur | Tx1: Group 1 – (Female (65.8%), Group 2 – Male (34.2%)  Tx2: Group 1 – Female (64.1%), Group 2 – Male (35.9%) | NR | Tx1: Primary (42.6%), Secondary (57.4%)  Tx2: Primary (46.2%); Secondary (53.8%) | | NR | NR | NA |
| Smith, 2015 | NR | NR | Tx1: Academic STEM faculty chosen to serve on faculty hiring committee  Tx2: Montana State University faculty | NR | NR | NR | | NR | NR | NR |
| Warren, 2017 | NR | Tx1: Minority Status (18.2%), Non-Minority Status (81.8%)  Tx2: Minority status (14.7%), Non-Minority status (85.3%) | Tx1: Full-time (83.6%), Part-time or Volunteer (16.4%)  Tx2: Full-time (88.2%), Part-time or Volunteer (11.8%) | Tx1: Group 1 – Minority (90.9%), Group 2 – Non-Minority (9.1%)  Tx2: Group 1 – Minority status (89.7%), Group 2 – Non- Minority Status (10.3%) | Tx1: Christian (Protestant) (34.5%), Christian (Catholic) (29.1%), Atheist/Agnostic (12.7%), Other (23.6%)  Tx2: Christian (Protestant) (39.7%), Christian (Catholic) (20.6%), Atheist/Agnostic (14.7%), Other (25.0%) | Tx1: High school/Associates/Some College (10.9%), College Graduate (41.8%), Graduate School (47.3%)  Tx2: High school/Associates/Some College (8.8%), College Graduate (54.4%), Graduate School (36.8%) | | Tx1: Less than $15,000 – $39,999 (12.7%), $40,000 – $54,999 (14.5%), $55,000 – $69,999 (12.7%), $70,000 – $84,999 (18.2%), $85,000 –$99,999 (12.7%), $100,000 or more (29.1%)  Tx2: Less than $15,000 –$39,999 (16.2%), $40,000 – $54,999 (19.1%), $55,000 – $69,999 (8.8%), $70,000 – $84,999 (17.6%), $85,000 –$99,999 (10.3%), $100,000 or more (27.9%) | NR | Tx2: Online written and video educational intervention |
| Webb, 2012 | Tx1 and Tx2: Ireland | NR | Tx1 and Tx2: Manager | NR | NR | NR | | NR | NR | NR |
| Wiseman, 1979 | NR | NR | Tx1 and Tx2: Heads of department and counselling personnel | Tx1: Group 1 – Female (33%), Group 2 – Male (67%)  Tx2: Group 1 – Female (27%), Group 2 – Male (73%) | NR | NR | | NR | NR | NR |
| Woolnough, 2007 | NR | Tx1: British – White (85%), British – Afro Caribbean (4%), Mixed race (4%), Indian (7.4%)  Tx2: British – White (96%), British – Afro Caribbean (0%), Mixed race (4%), Indian (0%) | Tx1: CPN (15%), Technician (4%), Nurse therapist (11%), Nurse specialist (15%), Team leader (7%), Lead nurse (7%), Night manager (4%), Ward manager (7%), Day unit manager (7%), Project lead (7%), CPA coordinator (11%), Care manager (4%)  Tx2: CPN (11%), Technician (4%), Nurse therapist (4%), Nurse specialist (11%), Nurse consultant (4%), Team leader (4%), Lead nurse (4%), Ward manager (15%), Day unit manager (4%), Project lead (7%), CPA coordinator (11%), Care manager (7%) | Tx1 and Tx2: Group 1 – Female (100%) | NR | Tx1: Diploma (15%), Advanced diploma (15%), Degree (56%), Masters (11%), MPhil (4%)  Tx2: Diploma (19%), Advanced diploma (15%), Degree (48%), Masters (19%) | | NR | NR | NR |
| **Companion paper*** | | | | | | | | | | |
| Huis, 2019  (1/2) | NR | NR | Tx1, Tx2, and Tx3: Female entrepreneur plus husband - 85% of the couples worked together as a couple in their business (e.g., agriculture, selling products) | Tx1, Tx2, and Tx3: Group 1 – Female (100%), Group 2 – Male (NA) | NR | NR | | NR | NR | NR |
| NA: Not Applicable  NR: Not Reported | | | | | | | | | | |
| ^1^O'Neill J, Tabish H, Welch V, Petticrew M, Pottie K, Clarke M, Evans T, Pardo Pardo J, Waters E, White H, Tugwell P. [Applying an equity lens to interventions: using PROGRESS ensures consideration of socially stratifying factors to illuminate inequities in health](http://www.jclinepi.com/article/S0895-4356(13)00334-X/abstract). Journal of Clinical Epidemiology. 2014, 67 (1), pg. 56-64. doi:10.1016/j.jclinepi.2013.08.00 | | | | | | | | | | |

# Appendix 14 – Definitions of Gender and Sex

| **Author, year of publication** | **Definition of Gender** |
| --- | --- |
| Bapna, 2021 | Gender as a variable is defined as man/woman, it is unclear how this was determined. Study uses sex and gender terms interchangeably; no explicit definition is provided. |
| Bates, 2019 | Self-identification of gender through a pre-intervention survey. Study uses sex and gender terms interchangeably.  “Social scientists investigating the concept of gender instead argue and empirical research shows that gender is a socially constructed facet of everyday life consistently reinforced through dominant gender norms such as expectations, interactions, organizational processes, and social institutions.” |
| Brady, 2015 (two studies) | Study uses sex and gender terms interchangeably across all studies within the article; no explicit definition is provided. |
| Bulte, 2017 | Study uses sex and gender terms interchangeably; no explicit definition is provided. |
| Chinen, 2017 | “Gender– a social construction regarding the roles of women, girls, men, and boys. Sex– which refers to the biological characteristics of being female or male.” |
| Cook, 2019 | Study uses sex and gender terms interchangeably; no explicit definition is provided. |
| Dahl, 2018 | Gender as a variable is defined as man/woman, it is unclear how this was determined. Study uses sex and gender terms interchangeably; no explicit definition is provided. |
| Ginter, 2020 | The terms women and female are used interchangeably, no explicit definition is provided. |
| Grisso, 2015 | Study uses sex and gender terms interchangeably; no explicit definition is provided. |
| Huis, 2019 | Study uses sex and gender terms interchangeably across all studies within this article; no explicit definition is provided. |
| Huis, 2019 | The intervention provided training on gender and gender equity. Study uses sex and gender terms interchangeably; no explicit definition is provided. |
| Ismayilova, 2017 | The intervention was related to education about gender norms and gender equality beliefs. Study uses sex and gender terms interchangeably; no explicit definition is provided. |
| Matsutaka, 2022 | Self identification of gender through a pre-intervention survey. Study uses sex and gender terms interchangeably; no explicit definition is provided. |
| O’Meara, 2018 | Self identification of gender through a pre-intervention survey. Gender and sex terms are used interchangeably throughout the study. A specific definition of either sex or gender is not provided. |
| Paek, 2022 | Gender was defined as a variable to be man/woman. Study uses sex and gender terms interchangeably; no explicit definition is provided. |
| Peterson, 2019 | Gender was defined as a variable to be male/female. Study uses sex and gender terms interchangeably; no explicit definition is provided. |
| Rivera, 2019 | Study uses sex and gender terms interchangeably; no explicit definition is provided. |
| Shankar, 2015 | Study uses sex and gender terms interchangeably; no explicit definition is provided. |
| Smith, 2015 | Study uses sex and gender terms interchangeably; no explicit definition is provided. |
| Warren, 2017 | Self identification of gender through a survey. Gender and sex terms are not conflated in the study.  “Cisgender refers to individuals whose gender identities and/or gender expression corresponds to their assigned biological sex.  Transgender, in contrast, refers to individuals whose gender identities and/or gender expressions differ from their assigned birth sex.” |
| Webb, 2012 | Study uses sex and gender terms interchangeably; no explicit definition is provided. |
| Wiseman, 1979 | “Gender is assigned at birth and a proper set of attitudes and behaviors are then expected and generally adopted.”  Study uses sex and gender terms interchangeably. |
| Woolnough, 2007 | Self identification of gender through a pre-intervention survey. Gender and sex terms are used interchangeably throughout the study. A specific definition of either sex or gender is not provided. |

# Appendix 15 – Intervention Characteristics

| **Intervention Category #1: Quantification of gender inequities in funding, publications, promotion and compensation** | | |
| --- | --- | --- |
| *Intervention sub-type* | *Number of studies reporting* | *Examples* |
| Reporting on the impact of gender equity efforts (made publicly available to ensure accountability) | 1 | - Transparent work activity is being published within departments to ensure shared rotation of administrative and service work in an academic environment to promote equity |
| **Intervention Category #2: Behavioural or systemic change** | | |
| *Intervention sub-type* | *Number of studies reporting* | *Examples* |
| Recognition of the systemic nature of gender inequity and the need for systemic solutions from organizations | 3 | - Recognizing the need of organizations (academia) to provide a solution to address faculty women and faculty from minority groups bearing the disproportionate burden of administrative, service, and mentorship tasks, resulting in less time spent on research. - Recognizing the role of unconscious and unintentional biases about race and the gender of university instructors when student’s complete student evaluations of teaching. - Acknowledging that men and women have equal rights to participate in, and benefit from, decision making about interventions to reduce greenhouse gas emissions. |
| Gender-inclusive language in recruitment, hiring, and grants and funding assessments | 2 | - Faculty recruitment aimed at increasing the number of women faculty in STEM at one US university where increasing diversity had historically proved elusive. - Hiring systems and diversity structures. |
| Use of quotas | 2 | - Gender quotas can help climate policy interventions to bring about increases not only in equality, but also in programme effectiveness raises questions about the reasons behind such effects. - Gender quotas for women in traditionally male dominated military environment to improve men’s bias towards women. |
| Gender bias training and champions of gender equity | 10 | - Program for Respect, Inclusion, and Diversity of LGBTQ Employees (PRIDE), a training program for human resource staff and health professionals to promote appropriate responses to the stress and harassment experienced by lesbian, gay, bisexual, transgender, and queer or questioning (LGBTQ) individuals in the workplace. - Investigating gender bias in student professor evaluations with identical excerpts from the transcript of a lecture and randomly assigned either a male or a female name to the instructor who had given the lecture. - A teacher-training program that aimed to promote positive gender socialization in Uganda, the program positively influenced teachers’ knowledge about the difference between gender and sex, and their attitudes toward gender roles and gender identity. - The Gender By Us® Toolkit, decreased implicit gender bias compared to a generic conversation. The Toolkit provides education about implicit bias and tools that raise awareness of gender inequality. Unique to this intervention, participants draw on personal experiences to engage in semi-structured conversations with each other to better understand how components of dominant gender structure influence their daily lives. - Three online interventions for aging-focused professionals designed to increase knowledge of TGNC-related terminology, decrease self-reported negative attitudes towards TGNC individuals, and increase self-efficacy for affirmative interactions. Interventions includes written educational information, a video demonstration, or both the written educational information and the video demonstration. - Adding language to student evaluations describing that women and instructors of colour are systematically rated lower in evaluations then white men. - Part of the intervention included providing an experiential workshop on implicit bias and the research on how it shapes divisions of labor in colleges and universities. - Focused on integrating women into the Norway military, the study surveyed recruits on how female integration to squads affects perceptions of gender roles and gender identity. - All members of the household participated in a gender sensitive family coaching session to raise awareness about child protection issues and address normative gender beliefs related to family violence. - Mentors were provided with gender and stereotyping in the workplace training. |
| **Intervention Category #3: Career flexibility** | | |
| *Intervention sub-type* | *Number of studies reporting* | *Examples* |
| Integrated career–life planning, coaching to create a customized plan to meet both career and life goals, and a time-banking system | 1 | - Addressing work-family conflict to support participants in both aspects of life. |
| Flexible policies, including family-friendly, parental and career flexibility policies | 1 | - Introducing flexible policies around schedule development. |
| **Intervention Category #4: Increased visibility, recognition and representation** | | |
| *Intervention sub-type* | *Number of studies reporting* | *Examples* |
| Career development planning | 6 | - Manuscript writing program to improve the total number of manuscript publications, and first author publications. - Business training was provided to improve outcomes for women or maritally owned/operated agricultural businesses. - Workshops increased the total number of publications and total number of federal grants in the treatment group. - Based on feedback from the participants a plan was put in place to obtain desired training tailored to their needs. - Access to culturally adapted training to improve women’s economic and social empowerment. - Female caregivers received livelihood planning, training, and coaching with trained field workers as part of a graduated approach to microfinance. |
| Leadership program | 3 | - Targeted agency-based empowerment training can significantly increase women’s capacity to engage effectively. The intervention is anchored in the core properties of individual agency and draws from basic tenets of positive psychology. It is aimed at increasing self-knowledge and developing actionable growth strategies. - As part of the intervention department teams were provided with training, tools and resources to create their work activity dashboards. Evidence-based policies and practices were shared, and departments could use these to proactively shape equitable workloads. Department teams took the lead to begin creating Department Equity Action Plans - Participation in the peer mentorship program led to a perceived increase in leadership effectiveness, more promotions, and significant improvement in personal development outcomes. |
| Ensure availability of role models to foster identity compatibility and belonging | 1 | - Participation in the mentorship workshops led to women who were more likely to stay in academia and more likely to receive tenure in prestigious institutions. |
| **Intervention Category #5: Creating opportunities for development, mentorship and sponsorship** | | |
| *Intervention sub-type* | *Number of studies reporting* | *Examples* |
| Career advising plan | 3 | - Female members attend weekly or monthly meeting and are able to exchange experiences and information about production and business, as well as enables the microfinance fund staff and external experts to disseminate knowledge on family, gender, and other issues. - Female borrowers were provided additional business training tailored to the needs of women. - Feedback was solicited to discover which types of training would be most beneficial to their needs and desires. |
| Peer mentoring program | 1 | - Eligible mentees and mentors applied to the “Challenging Perceptions” program to be paired for 12 months in an intervention specifically designed to help the nurses break the “glass ceiling”. |
| **Intervention Category #6: Financial support** | | |
| *Intervention sub-type* | *Number of studies reporting* | *Examples* |
| Financial support | 4 | - Partnering with the largest microfinance fund in Northern Vietnam by providing women with loans, savings opportunities, and mutual assistance funds to improve the quality of life of the women and their families. - Female caregivers were given access to seed capital grants to jump-start or expand livelihood activities. - Female borrowers were given access to micro-credit loans. - Female borrowers were given access to microfinance initiatives and supported with training to improve their income generating activity. |

# Appendix 16 – Details on intervention outcomes and results

| **Author, year** | **Intervention** | **Comparator** | **Primary Outcome** | **Abstract conclusion** | **Does the intervention focus on the individual, the organization, or the system?** |
| --- | --- | --- | --- | --- | --- |
| **Included trials** | | | | | |
| Bapna, 2021 | The Search List intervention was designed to lower search barriers by minimizing intermediaries between subjects and other conference participants. Individuals in this treatment condition received an email from the conference organizer with a list of 16 target contacts, presented as “networking recommendations.” Targets were randomly selected from among those in the same treatment condition and were not reciprocal—if A received B as a recommendation, B did not necessarily receive A. Put differently, B received a list of 16 recommendations that did not necessarily overlap with A’s recommendations. Thus, A was one hop (i.e., network “step”) away from 16 people (A’s direct recommendations) but two hops away from about 256 individuals (the individuals who appeared on the lists that were sent to A’s direct recommendations). Through their recommendations—and the recommendations of their recommendations—subjects were indirectly connected to many other conference participants (e.g., A’s recommendations could introduce them to their own recommendations). Thus, through this intervention, the network distance between individuals in the Search List condition is likely to be reduced. The left-side panel of Figure 1 shows the network structure of recommendations for the Search List treatment condition. Two networking barriers—search and social—differentially affect men and women. The search intervention was designed to facilitate locating diverse contacts and information.  Study included/randomized 958 participants. | Individuals in the Control group received a generic email prompting them to network with others at The Conference. | Number of new contacts met.  Number of LinkedIn connections.  Average minutes spent talking to each new contact.  Odds of changing jobs. | POSITIVE  Their findings indicate that the search intervention increased the number of new contacts women met by 57%, the time they spent talking with them by 90%, the number of LinkedIn connections they added by 29%, and their odds of changing jobs by a factor of 1.6. The social intervention also increased the time women spent talking to new contacts by 66%. However, the interventions did not improve men’s outcomes. Their results show that simple interventions can help women expand their networks and find jobs. | Individual |
| Bates, 2019 | The intervention group participated in conversation specifically related to gender using the Gender By Us® Toolkit. The Toolkit provides step-by-step instructions to guide a semi structured conversation. The first step prior to initiating the conversation is to review the Toolkit’s conversation “Do’s and Do Not’s” to promote a safe environment for conversation. The second step involves facilitators providing basic education regarding the terms “gender norms” and “implicit bias,” followed by a group discussion regarding gender roles and stereotypes using the “Man/Woman Box” icebreaker activity. From there, the intervention involves small groups self-facilitating a conversation guided by data point and prompt cards from the Toolkit.  Study included/randomized 23 participants. | Generic conversation. | (1) the  Social Dominance Orientation Scale, (2) the Neosexism Scale, and (3) the Modern Sexism Scale | POSITIVE  Quantitative results showed the intervention group scored significantly higher on the Modern Sexism Scale at post-test than the control group (p ¼ .00), demonstrating increased acknowledgment of gender discrimination. Both groups also were asked open-ended questions related to the intervention and implicit gender bias. Qualitative results suggested that participants’ overall perception was that the intervention increased their awareness of implicit gender biases within themselves and their ability to identify biases in others, yet a comparatively low number of intervention participants retained the ability to identify an example of implicit gender bias at post-test. Findings may indicate the intervention had a small but positive effect on disrupting implicit gender bias. | Individual |
| Brady, 2015  (Two studies are reported in this article. This summary refers to the first study). | Participants first read an identical profile of a fictitious investment company. Depending on random assignment, they then read either that the company had a diversity training program (“Fostering Women's Success”) or a general managerial training program (“Fostering Employee Success”). These program descriptions were identical except that the diversity training program included phrases specifically about improving women's outcomes in the company. After reading the program description, participants wrote a one-sentence summary of the program. Participants then learned that they would have 2.5 min to review a list of applicants who had applied for a managerial position at the company. The list contained 20 male and 20 female applicants, listed by first name and last initial to ensure that applicant gender was salient. The document also provided applicants' qualifications (i.e., years of relevant work experience, highest degree earned, score on an employment test ranging from 0 to 30, and score from a human resources resume screening ranging from 0 to 5). Male and female applicants were matched such that for every male applicant, there was a female applicant with identical qualifications. Next, participants were given 1 min to review the short list of applicants who the company had selected for interviews.  The shortlist included the top seven male applicants and top 3 female applicants. Thus, despite being equally qualified and equally represented in the applicant pool, women were interviewed at only 43% the rate of men.  Study included/randomized 119 participants. | Fostering Employee Success | Perceived the company as more procedurally for women.  Perceptions of disparate treatment. | NEGATIVE  In Experiment 1, even when a company's hiring decisions disadvantaged women, women perceived the company as more procedurally just for women and were less supportive of sexism litigation when the company offered diversity training, compared to when it did not. Additionally, these effects were most pronounced among women who endorsed benevolent sexist beliefs and mitigated among those who rejected benevolent sexist beliefs. Together, these experiments demonstrate that diversity structures can make it difficult for women to detect and remedy discrimination, especially women who hold benevolent sexist beliefs. | Organizational |
| Brady, 2015  (Two studies are reported in this article. This summary refers to the second study). | Participants read an article formatted to resemble a New York Times contribution describing a woman's lawsuit against her employer, Novartis Pharmaceuticals (see Appendix B). The woman claimed that Novartis discriminated against her in pay and promotions because of her gender. She alleged that she was paid $105 less per month than male employees in comparable positions and was passed over for promotion by men with inferior sales numbers. In the diversity structure condition, the article included a sentence stating that a lawyer for Novartis denied the claim and stated that the company had been recognized by Diversity Careers magazine as a top company for women. In the control condition, the lawyer also denied the claim, but did not mention the diversity award. The remaining information was identical between conditions.  Study included/randomized 178 participants. | Control – no mention of a diversity award. | Procedural justice.  Perceived discrimination. | NEGATIVE  In Experiment 3, women perceived a company as more procedurally just and less discriminatory when the company had been recognized for positive gender diversity practices compared to when it had not. Additionally, these effects were most pronounced among women who endorsed benevolent sexist beliefs and mitigated among those who rejected benevolent sexist beliefs. Together, these experiments demonstrate that diversity structures can make it difficult for women to detect and remedy discrimination, especially women who hold benevolent sexist beliefs. | Individual |
| Bulte, 2017 | Gender and business (G&B) training intervention offered to female clients only of TYM. The trainings provided through TYM fund are based on the Gender and Entrepreneurship Together (GET) Ahead for Women in Enterprise Training Package and Resource Kit, designed by the International Labour Organization (ILO). The standard content has been slightly modified to better fit the Vietnamese context.  The first module of the program covered basics on gender and entrepreneurship, promotion of equality between men and women, and the life cycle of people and enterprises. The second module considered the businesswoman and her self-confidence. The third module focused on the businesswoman and her environment,  self-development, and business mapping.  The fourth module discussed business projects, including business ideas, opportunities, and challenges. The fifth module covered marketing and sales. The sixth module covered calculations and aspects of financial literacy. The seventh module focused on managing cash. The eighth module discussed how to record accounts receivable and accounts payable. The ninth and final module covered how to calculate cost of production and cost of goods sold.  Study included/randomized 4041 participants. | Gender and business training where male partners were invited to join. | Business knowledge, general practices, innovation, marketing, record keeping and planning, sales, and profit | POSITIVE  They find evidence of economically substantive impacts on knowledge, practices, and outcomes, and on the extensive margin (entry and exit). They also document that it takes time for the “downstream” outcomes of the trainings to materialize; although they find evidence of medium-term effects, no such evidence exists for the short term. Inviting husbands to participate in the trainings does not affect any of their knowledge or practice measures, but they document weak evidence for differential impact on (agricultural) sales and profits. | Individual |
| Chinen, 2017 | The training was organized in two stages. First, the implementing partners provided a three-day training of trainers in March 2015 for the coordinating center tutors (CCTs), district school inspectors, and MoESTS personnel. The training explained theoretical concepts of gender, conflict, and peacebuilding using participatory approaches, such as role playing, discussions, and storytelling, that incorporated familiar examples from Karamoja. Second, trained CCTs and inspectors delivered a three-day training for one thousand teachers at central locations in five districts of Karamoja.1 The training aimed to empower primary teachers as agents of change, promote positive models of masculinity and femininity, and redress gender biases and question social norms. Moreover, the training aimed to create awareness of alternative norms and practices related to gender equality, build skills to help engage pupils in constructive dialogue, and provide materials to foster a shift in gender attitudes and beliefs and promote gender-sensitive practices in the classroom (Development Research and Training 2015). In August/September and November 2015, teachers received refresher trainings to reinforce content.  Study included/randomized 916 participants. | Training + Reinforcing Text Messages Group, Control Group | Knowledge about the difference  between gender and sex  Knowledge about gender, identity,  and conflict  Attitudes toward gender roles,  Index 1-3  Attitudes toward gender identity and equality  Gender-responsive and peaceful  self-reported practices  Gender equality self-reported  practices | NEUTRAL  Their study found evidence that the program positively influenced teachers’ knowledge about the difference between gender and sex, and their attitudes toward gender roles and gender identity. They found no quantitative evidence for any short-term change in teachers’ practices as a result of the program, nor did they find quantitative evidence of effects from a complementary, randomly assigned text-message intervention meant to reinforce the information delivered during the training. Qualitative research suggested that, while teachers adopted basic practices taught in the training, they were unready or unable to adopt more complex practices. The main implication is that training can influence teachers’ knowledge and attitudes on gender equality,  but traditional gender norms can be a barrier to changing behavior in the short term. | Individual |
| Cook, 2019 | Under the gender quota treatment, their field team ensured that at least 50% of the participants in each group assigned to the treatment condition were women… Participants played the game in groups of eight, and their framing described the experiment as a decision-making activity in which participants would make decisions about harvesting from a shared local forest. The treatment and control groups in each village played the game sequentially; however, in each village their field teams randomized the order of implementation. Across the treatment and control conditions, the basic structure of the game was designed so that participants faced trade-offs between collectively conserving the resource in pursuit of a shared PES incentive and free-riding to profit from the resource at the expense of other group members who contributed to its conservation.  Study included/randomized 440 participants. | No gender quota. | Choice of a woman as a leader.  Share of communication by women.  Proportion of females in the group. | POSITIVE  Groups with the gender quota conserved more trees as a response to a ‘payment for ecosystem services’ intervention and shared the payment more equally. They attribute this effect to the gender composition of the group, not the presence of female leaders. | Individual |
| Dahl, 2018 | Females were assigned randomly to rooms, with 2 females per room unless there was an odd number, in which case 3 women were assigned to a room. Finally, each room was randomly assigned male soldiers up to the specified room size. Since the number of individuals in a troop does not necessarily equal a multiple of 6, the troop officer could make manual adjustments to even out room sizes.  Study included/randomized 900 participants. | No intervention, no females are assigned to rooms. | Gender attitudes after bootcamp.  Female leadership attitudes.  Gender attitudes after 6 months of service. | POSITIVE  They find that living and working with women for 8 weeks causes men to have more egalitarian attitudes. There is a 14-percentage point higher fraction of men who think mixed-gender teams perform as well or better than same-gender teams, an 8-percentage point increase in men who think household work should be shared equally and a 14 percentage point increase in men who do not completely disavow feminine traits. Moreover, men exposed to  mixed-gender teams are more likely to choose military occupations immediately after boot camp which have a higher fraction of females in them. But these effects do not persist once treatment stops. Treated men’s attitudes converge to those of the controls in a 6-month follow up survey and there is no long-term effect on choosing fields of study, occupations or workplaces with a higher fraction of women in them after military service ends. Contrary to the predictions of many policymakers, they do not find that integrating women into squads hurt male recruits’ performance or satisfaction with service, either during boot camp or their subsequent military assignment.  These findings provide evidence that even in a highly gender-skewed environment, gender stereotypes are malleable and can be altered by integrating members of the opposite sex. But they also suggest that without continuing intensive exposure, effects are unlikely to persist. | Individual |
| Ginter, 2020 | The national CeMENT workshops were designed to expose participants to role models (senior female economists), to transmit information about what it takes to be successful, and to build peer networks of female junior faculty working in similar research areas.  Study included/randomized 365 participants. |  | Tenure stream job alone, in top 100, in top 30 ranked institution.  Tenure alone, in top 100, top 50, top 30 ranked institution.  Number of publications and grants. | POSITIVE  Results indicate that relative to women in the control group, treated women are more likely to stay in academia and more likely to have received tenure in an institution ranked in the top 30 or 50 in economics in the world. | Individual |
| Grisso, 2015 | The three-tiered intervention included components that were aimed at (1) the professional development of women assistant professors, (2) changes at the department/division level through faculty-led task forces, and (3) engagement of institutional leaders over 3 years.  Study included/randomized 134 patients. | No intervention | Number of publications and number and amount of grants. | POSITIVE  Academic productivity and work self-efficacy improved significantly over the 3-year trial in both intervention and control groups, but the improvements did not differ between the groups. Average hours worked per week declined significantly more for faculty in the intervention group as compared with the control group (-3.82 vs. -1.39 hours, respectively, p = 0.006). The PhD faculty in the intervention group published significantly more than PhD controls; however, no differences were observed between MDs in the intervention group and MDs in the control group. | Organizational |
| Huis, 2019 | Huis et al., invited female microfinance borrowers and their husbands to the training as both parties should be involved to change existing gender roles with respect to their income‐generating activity.  Huis et al., investigated the impact of the training on goal‐setting skills, self‐esteem, and the couples’ interaction in a subsequent task. In two field experiments, female borrowers and their husbands were randomly assigned to one of three conditions: (a) goal‐setting training and setting goals as couple, (b) goal‐setting training, and setting goals individually, or (c) no training (control condition).  Study included/randomized 74 couples. | Goal‐setting training and setting goals individually.  No training (control). | Goal setting.  Self esteem.  Couple interaction – women’s empowerment. | POSITIVE  Participation in the training increased women's SMART (specific, measurable, achievable, realistic, time bound) goal‐setting skills.  Descriptively, they found some initial evidence of increased women's empowerment in the  interaction (Study 2). | Individual |
| Huis, 2019 | Female borrowers received access to training for which their husbands were also invited OR individually.  Female borrowers in the two treatment arms were invited to nine monthly training sessions that lasted 45–60 min each. The training sessions were based on the GET (Gender and Entrepreneurship Together) Ahead program.  Study included/randomized 4041 participants. | A control group of female borrowers where they received a loan only. | Women’s personal beliefs score.  Women’s experience of relational friction.  Women’s experience of relational oppression.  Women’s intra-household decision making power.  Women’s decision making about larger expenditures.  Women’s decision making in the daily domain. | POSITIVE  They found that the GET Ahead training improves women’s empowerment on all three aspects: increased control beliefs and intra-household decision making power (only on larger expenditures) and decreased relational friction.  However, the results on relational frictions should be taken with care due to possible underreporting.  Moreover, in general, they found no additional impacts of inviting husbands to the training. Finally, their results suggest that it takes some time before the training starts to improve women’s empowerment. They observe no short-term but only mid-term effects from before the training to 12 months after the training. | Individual |
| Ismayilova, 2017 | The package of economic and livelihood interventions (TU arm) engaged female  caregivers as the primary beneficiaries and included (a) savings group formation and training using the Village Savings and Loan  Association (VSLA) model; (b) livelihood planning and household management training; (c) seed capital grants to jump-start or  expand livelihood activities; and (d) one-on-one mentoring and coaching on livelihood development conducted by trained field workers.  In addition to the economic  intervention, the study tested an added gender sensitive family coaching component (TU Plus arm), which was primarily intended to raise the awareness of all members of the household about the context specific child protection issues (e.g., early and forced marriage, girl’s education, child labor and labor-related child separation) and  address normative gender beliefs related to family violence and wives’ role in family decision making (e.g., wife’s contribution to  household economy and decisions affecting child’s future).  Study included/randomized 360 participants. | No intervention (waitlist) | Physical violence  Emotional violence  Quality of marital relationship.  Decision making power.  Gender equality beliefs.  Financial autonomy. | POSITIVE  Compared with the control group, there was a significant improvement in both the TU arm and the TU Plus arm in women’s financial autonomy and in quality of marital relationships. In addition, women in both intervention arms reported a significant reduction in emotional spousal violence in the past year, with the effect size greater for the combined intervention | Individual |
| Matsutaka, 2022 | Program for Respect, Inclusion, and Diversity of LGBTQ+ Employees (PRIDE), a training program for human resource staff and health professionals to promote appropriate responses to the stress and harassment experienced by lesbian, gay, bisexual, transgender, and queer or questioning (LGBTQ+) individuals in the workplace.  Study included/randomized 39 participants. | No intervention. | Knowledge about LGBTQ+ people, subjective comprehension of LGBTQ+ people, homophobia, transphobia, implicit homophobia and transphobia, self-efficacy when responding to and providing support to LGBTQ+ people. | POSITIVE  The results suggest PRIDE may increase knowledge regarding LGBTQ+ people, subjective comprehension of LGBTQ+ people, and self-efficacy when responding to and providing support to LGBTQ+ people and reduce explicit homophobia and transphobia among occupational health staff and human resource staff with the potential to provide LGBTQ+ consultation services. | Individual |
| O’Meara, 2018 | A theory-driven, 18-months intervention, targeting routine work practices, department conditions, and the readiness of faculty to intervene to shape more equitable outcomes. The intervention involved:  (a) a workshop on implicit bias and how it can shape divisions of labor  (b) arming department teams with tools to create and display faculty workload activity dashboards  (c) using dashboards to identify equity issues and sharing work practices and policies to mitigate bias and proactively design for equity, and  (d) an optional professional development webinar series on aligning time and priorities as a faculty member.  Study included/randomized 472 participants. | No intervention. | Department conditions.  Work practices.  Action readiness.  Perception of fairness.  Self-advocacy. | POSITIVE  Post intervention faculty in participating departments were more likely than before the intervention to report work practices and conditions that support equity and action readiness in their department, and that teaching and service work in their department is fair. Participating  departments were significantly more likely than control departments to report practices and  conditions that support equity and greater action readiness to address issues of workload equity in their department. Finally, participating department faculty were more likely than control department faculty to report increased self-advocacy and were more likely than control department faculty to report that the distribution of teaching and service work in their department is fair. | Individual / Organizational |
| Paek, 2022 | The intervention was designed to increase supervisor support for work-family integration and to increase employees’ perceptions of control over their work time. The three components of STAR/T were more clearly defined: participatory sessions, employee outside activities, and separate training for supervisors. STAR/T was designed to take place over a 4-month period of time and the facilitators would guide employees and supervisors through the face-to-face, participatory sessions using structured and interactive activities (including role plays, games, etc.). The participatory sessions and outside activities were sequenced to build on each other and to reinforce the lessons learned.  Study included/randomized 209 participants. | No intervention. | Concern over control of work schedule | POSITIVE  Results show that the workplace initiative significantly reduced concerns managers had about schedule control. In models stratified by gender, the  initiative’s effect was statistically significant among male managers, but not among female managers. Male managers tended to have greater concerns about schedule control than female managers, but male managers who received the  intervention reduced their level of concern to the level of female managers who did not receive the intervention. | Individual / Organizational |
| Peterson, 2019 | In the treatment condition, the solicitation and the evaluation instrument used language that they expected to mitigate gender biases. The added language was: “Student evaluations of teaching play an important role in the review of faculty. Your opinions influence the review of instructors that takes place every year. Iowa State University recognizes that student evaluations of teaching are often influenced by students’ unconscious and unintentional biases about the race and gender of the instructor. Women and instructors of colour are systematically rated lower in their teaching evaluations than white men, even when there are no actual differences in the instruction or in what students have learned. As you fill out the course evaluation, please keep this in mind and make an effort to resist stereotypes about professors. Focus on your opinions about the content of the course (the assignments, the textbook, the in-class material) and not unrelated matters (the instructor’s appearance).”  Study included/randomized 247 participants. | In the control condition, students received the standard student evaluations of teaching (SET) survey for their department. | Overall evaluation for the teacher.  Overall evaluation for the course.  Teaching effectiveness. | POSITIVE  Students in the anti-bias language condition had significantly higher rankings of female instructors than students in the standard treatment. There were no differences between treatment groups for male instructors. These results indicate that a relatively simple intervention in language can potentially mitigate gender bias in student evaluation of teaching. | Individual |
| Rivera, 2019 | A survey experiment in which they presented all participants with an identical lecture transcript but randomly varied instructor gender and the number of points on the rating scale used in evaluations (10 points vs 6 points scale).  Study included/randomized 369 participants. | Male instructor x female instructor  Perceived male instructor x perceived female instructor.  10-point scale x 6 point scale | Perceived brilliance, average brilliance, 10-point scale mean rating, 6 point scale mean rating. | POSITIVE  Results suggested that the number of scale points affects the extent to which gender stereotypes of brilliance are expressed in quantitative ratings. These results highlight how seemingly minor technical aspects of performance ratings can have a major effect on the evaluation of men and women. | Individual |
| Shankar, 2015 | The agency-based empowerment training builds upon a 4-day workshop developed by the Empowerment Institute, based in New York, where individuals participate in an introspective examination of key areas of their life. The training focuses on the following competencies: (a) exercises are relevant and meaningful to the individual; (b) the locus of control sits firmly within the individual rather than on external forces; (c) a simple process of cognitive reframing is adapted; and (d) participants self-reflect to assess progress toward their goals. Over the course of the workshop, through individual and interactive exercises, participants examine aspects of their emotions, relationships, their health=body, money, and work. In this training, exercises and examples were designed to support specific challenges faced in new entrepreneurial endeavours.  Study included/randomized 300 participants. | Entrepreneurial Training | Improved cookstove sales | POSITIVE  The empowerment training led to more than doubling of sales for both genders. In addition, participants in the intervention group were significantly more likely to  demonstrate business commitment over time and nearly three times more likely to be higher sellers (relative risk¼2.7, 95% CI [1.4, 5.4]), controlling for gender and rural=urban locale. Women outsold men by a margin of nearly 3 to 1 and were more likely to continue to pursue leads despite limited sales. Nonactive participants (those selling 1 improved cookstove or less) were a larger percentage of the control group (72%) than the intervention group (50%), and more men were nonactive participants (65% of men) compared with women (56% of women). These data show that women can serve as active improved cookstove entrepreneurs in both urban and rural settings and that targeted agency-based empowerment training can significantly increase women’s capacity to engage effectively within the improved cookstove value chain. | Individual |
| Smith, 2015 | The search committees randomly assigned to the intervention condition were contacted by email from a faculty peer with an invitation inviting them to participate in the (voluntary) training. In a small group setting, from 3-6 people, each randomly selected search committee received the three step intervention from a faculty peer: 1) an explanation of the printed search toolkit and a summary two-page “tip sheet” 2) a 20-30 minute overview of “unintentional” (i.e., implicit) gender-bias research, including a copy of a scientific article on the topic, and 3) an introduction to the importance of work-life integration (e.g., partner, family, and recreational support) when recruiting candidates and a recommendation to the search chair that all on-campus interview candidates connect with a faculty Family Advocate for a brief 15-minute confidential meeting to answer questions and discuss work-life integration practices at the university. Intervention groups also received a data sheet outlining faculty gender diversity figures and trends in their department compared to national standards and the percentage of PhDs awarded nationally to women in that field.  Study does not report the number of included/randomized participants. |  | Number of women short listed or phone interviewed.  Number of women among finalists.  Percent of offers made/accepted by a woman candidate. | POSITIVE  Results show that the numbers of women candidates considered for and offered tenure-track positions were significantly higher in the intervention groups compared with those in controls. Searches in the intervention were 6.3 times more likely to make an offer to a woman candidate, and women who were made an offer were 5.8 times more likely to accept the offer from an intervention search. | Individual |
| Warren, 2017 | Written educational group. Participants were provided with a brief series of paragraphs to review. For individuals randomized to this condition, the written information appeared automatically once they entered the intervention. The information provided relevant definitions. In addition, this handout provided a brief explanation about the importance of using clients’ preferred names and pronouns as well as how to move through the conversation if an incorrect pronoun or name is accidentally used.  Video group. Participants who entered this condition watched a brief video (approximately 2 minutes) that appeared and played automatically once participants entered the intervention. The video portrayed a role-played interaction between a provider and TGNC older adult for whom there existed a discrepancy in current name/gender and name/gender on previous records. The interaction provided comparable information to the written educational condition regarding definitions, pronoun and name usage, and instructions on how to conversationally apologize when incorrect pronouns and names are used.  Study included/randomized 155 participants. | Video group.  Written and video group combined. | Increased knowledge of TGNC related terminology  Decrease in self-reported negative attitudes towards TGNC individuals.  Increase in self-efficacy for positive interactions. | POSITIVE  Results showed no difference between interventions; participants in both conditions demonstrated a decrease in anti-TGNC attitudes and an increase in self-efficacy. | Individual |
| Webb, 2012 | Webb et al., examined the association between women and subordinate job roles versus men and superordinate job roles among managers with responsibility for company personnel selection decisions. Participants completed a gender-subordinate/superordinate role IAT under implementation intention versus standard IAT instructions.  Study included/randomized 40 participants. | IAT with Control measures | Effect of gender job role on explicit attitude measures  Effect of intervention and an interaction term on explicit attitude scores | POSITIVE  As expected, managers without a plan evidenced a strong implicit association between males and superordinate job roles and between women and subordinate job roles.  Thus, the ‘think manager–think male’ association originally identified by Schein (1973)  was prevalent among participants in the present research. However, the responses of managers who formed an implementation intention were indicative of a much weaker implicit bias for associating men with superordinate, and women with subordinate, job roles. Furthermore, the effects of implementation intention formation on implicit responses were maintained 3 weeks later even though participants were given no additional if–then instructions during the follow-up tests. Although it is possible that  participants simply remembered the initial instructions at follow-up this speaks to the value of implementation intention instructions over time. These findings therefore demonstrate that the modification of implicit responses via implementation intentions is both effective and durable. | Individual |
| Wiseman, 1979 | Two workshops  Workshop I (Summary)  A combination of lectures, large group participation, small group interaction and. Individual question and answer sessions comprised the format and approach to this learning experience.  Four major sessions took place at this workshop:  1. Lecturette on the topic of "Organizational Renewal".  2. Future projections (10 to 20 years from the present).  3. Modes of Communication - male/female.  4. Role play exercise.  Workshop II (Summary)  The workshop was designed to be more group oriented than the first workshop. A series of exercises took place with expectation that much group interaction would result.  Five major activities took place at this workshop:  1. "I wish exercise".  2. Career choice activity.  3. Advisor exercise.  4. Job sharing proposal.  5. Strategy for change.  Study included/randomized 49 participants. | No intervention | Attitude towards women scale | POSITIVE  Three administrations of the Attitude Toward Women Scale, sixty-two (62) completed forms of the experimental instrument. Advisor- Student Rating Scale and participant workshop evaluations were used to assess change.  The results of this study indicate that no significant differences existed among the three age groups identified. Only one significant difference emerged between the experimental and control group and that was Factor VI, Marital Relationships and Obligations, with the control group becoming more liberal.  Significant differences occurred between male and female subjects with females significantly more liberal on Factor II, Freedom and Independence, Factor III, Dating, Courtship and Etiquette, and Factor V, Sexual Behavior. | Individual |
| Woolnough, 2007 | The Challenging Perceptions 12-month, multi-faceted career development and mentoring programme was specifically designed to aid female mental health participants in their attempts to break the glass ceiling, which can be apparent within senior levels of the UK National Health Service. This study aimed to investigate the effects of a career development and mentoring programme on female mental health nurses’ career and personal development. ... "Mentee participants experienced an array of training and development events over the 12-month programme (e.g. action learning sets, gender awareness training), the central feature being the mentoring relationship, where mentees were matched with a senior figure who volunteered to become a mentor (such as the director of finance, director of nursing, executive director) from within their own NHS organization. The blend of intensive training and development in relation to mentoring, career development and glass ceiling issues made the programme unique. The programme was also designed to affect mentors based on the premise that mentoring is a reciprocal relationship."  Study included/randomized 54 participants. | No intervention. | Promotions  Role enrichment.  Career satisfaction.  Increased self-confidence.  Increase self-esteem. | POSITIVE  Results indicated that fifteen (55.5 per cent) mentees were promoted over the course of the study compared to seven (25.9 per cent) members of the control group. Other career advances for mentees were related to role enrichment and career satisfaction. Personal outcomes for mentees compared to the control group over the course of the study included increased self-confidence/self-esteem and increased satisfaction with their ability to deliver quality patient care. Mentors benefited from the experience in terms of an increased understanding of the mentoring role, increased connection with clinical staff and a desire to implement organisational change. | Organizational |
| **Companion paper*** | | | | | |
| Huis, 2019 | Huis et al., invited female microfinance borrowers and their husbands to the training as both parties should be involved to change existing gender roles with respect to their income‐generating activity.  Huis et al., investigated the impact of the training on goal‐setting skills, self‐esteem, and the couples’ interaction in a subsequent task. In two field experiments, female borrowers and their husbands were randomly assigned to one of three conditions: (a) goal‐setting training and setting goals as couple, (b) goal‐setting training, and setting goals individually, or (c) no training (control condition).  Study included/randomized 68 couples. | Goal‐setting training and setting goals individually.  No training (control). | Goal setting.  Self esteem.  Couple interaction – women’s empowerment. | POSITIVE  Participation in the training increased women's SMART (specific, measurable, achievable, realistic, time bound) goal‐setting skills. | Individual |

# Appendix 17 – Outcomes Examined in Included Studies (*from most to least frequent*)

| **Type of outcomes** | **Outcome counts** | **Number of studies reporting the outcome** |
| --- | --- | --- |
| **Microfinance**  (e.g., business knowledge, sales total, profit total, goal setting, self-esteem, etc.) | 69 | 5/24 |
| **Addressing bias / changes in biases**  (e.g., implicit homophobia and transphobia, neosexism scale, decrease self-reported negative attitudes towards trans- or gender non-confirming (TGNC) individuals, etc.) | 53 | 5/24 |
| **Workplace culture**  (e.g., work self-efficacy change, hours worked per week, perception of fairness in the workplace, likely to say the company addresses gender diversity issues, etc.) | 52 | 4/24 |
| **Gender equity**  (e.g., number of women short listed or interviewed, gender attitudes, female leadership attitudes, percent of offers accepted by a woman candidate, etc.) | 30 | 5/24 |
| **Academic workforce**  (e.g., probability of having a tenure stream job in a top 100/30/10 institution, probability of having a tenured position, overall teaching evaluation, etc.) | 16 | 2/24 |
| **Education**  (e.g., knowledge and subjective comprehension of the subject matter, increased knowledge in TGNC related terminology, etc.) | 12 | 3/24 |
| **Networking**  (e.g., number of new contacts met, average minutes spent talking with each new contact, number of LinkedIn connections, etc.) | 12 | 1/24 |
| **Academic output**  (e.g., number of publications total, peer reviewed, and first authored, number of pre-tenured grants, number of publications based on journal ranking, etc.) | 10 | 2/24 |

# Appendix 18 – Patient Partner Lay Summary.
